# Multiscale Modelling, Analysis and Simulation of Cancer Invasion Mediated by Bound and Soluble Enzymes

**DOI:** 10.1007/s11538-025-01535-w

**Published:** 2025-10-03

**Authors:** Mariya Ptashnyk, Chandrasekhar Venkataraman

**Affiliations:** 1 Department of Mathematics, Heriot-Watt University, The Maxwell Institute for Mathematical Sciences, Edinburgh, Scotland UK; 2 Department of Mathematics, University of Sussex, Sussex, UK

**Keywords:** Cancer invasion, Bound and soluble matrix-degrading enzymes, Multiscale modelling

## Abstract

We formulate a cell-scale model for the degradation of the extra-cellular matrix by membrane-bound and soluble matrix degrading enzymes produced by cancer cells. Based on the microscopic model and using tools from the theory of homogenisation we propose a macroscopic model for cancer cell invasion into the extra-cellular matrix mediated by bound and soluble matrix degrading enzymes. For suitable and biologically relevant initial data we prove the macroscopic model is well-posed. We propose a finite element method for the numerical approximation of the macroscopic model and report on simulation results illustrating the role of the bound and soluble enzymes in cancer invasion processes.

## Introduction

Invasion is one of the hallmarks of cancer (Hanahan and Weinberg 2000, 2011; Hanahan 2022). It is a complex process involving numerous interactions between cancer cells and the extracellular matrix (cf. the tumour microenvironment) facilitated by matrix degrading enzymes. Along with active cell migration, both individual and collective, and increased/excessive proliferation, these processes enable the local spread of cancer cells into the surrounding tissue. Any encounter with blood or lymphatic vessels (cf. tumour-induced angiogenesis, lymph-angiogenesis) in the tumour microenvironment offers the opportunity of intravasation, which together with subsequent extravasation, determines the dissemination of the cancer to secondary locations in the host, i.e., metastasis or metastatic spread. Further details of the invasion-metastasis process, and also extensive biological/clinical references, can be found in, e.g., Fidler (2003); Friedl and Wolf (2003); Hanahan and Weinberg (2000, 2011); Klein (2008); Talmadge and Fidler (2010).

Mathematical modelling of cancer invasion can be traced back to the model formulated in Gatenby (1995) using an approach from theoretical ecology and a modified predator-prey system, which focussed on the interactions between cancer cells and normal, healthy cells competing for space and other resources within a tissue. Spatial models of cancer invasion were then developed using systems of reaction-diffusion-taxis equations (Orme and Chaplain 1996; Gatenby and Gawlinski 1996; Perumpanani et al. 1996), focussing on haptotaxis as the key cell migration mechanism. A hybrid discrete-continuum approach was developed by Anderson et al. (2000) which enabled the depth of penetration of the ECM by individual cancer cells to be studied. The reaction-diffusion-taxis equations formulated in Anderson et al. (2000) were analysed in Marciniak-Czochra and Ptashnyk (2010). The effects of cell-cell and cell-matrix adhesion were incorporated in Gerisch and Chaplain (2008) using a non-local model. A number of studies considered the derivation of mixture or multi-phase models for tumour growth (Byrne et al. 2003; Franks and King 2003). The phase-field modelling approach to describe tumour growth and invasion was proposed in Cristini et al. (2009); Fritz et al. (2019); Fritz (2023) where the Cahn-Hilliard equation was used to model the invasion dynamics. An analysis of a diffusive interface model for tumour growth describing evolutions driven by long-range interactions was considered in Scarpa and Signori (2021), while the Cahn-Hilliard-Darcy model for tumour growth including nutrient diffusion, chemotaxis, active transport, adhesion, apoptosis and proliferation was derived in Garcke et al. (2016) with extensions to account for viscoelastic effects considered in Garcke et al. (2022). A recent review of mathematical models of cancer invasion can be found in Sfakianakis and Chaplain (2021).

A crucial part of the invasive/metastatic process is the ability of the cancer cells to degrade the surrounding tissue or extracellular matrix (ECM). The ECM is a complex mixture of macromolecules, some of which, like the collagens, are believed to play a structural role and others, such as laminin, fibronectin and vitronectin, are important for cell adhesion, spreading and motility. All of these macromolecules are bound within the tissue, i.e., they are non-diffusible. The extracellular matrix can also sequester growth factors and itself be degraded to release fragments which can have growth-promoting activity. Thus, while extracellular matrix may have to be physically removed in order to allow a tumour to spread or intra- or extravasate, its degradation may in addition have biological effects on tumour cells. Degradation of the ECM is achieved through the proteolytic activity of various matrix degrading enzymes (MDE). The experiments of Sabeh et al. (2009) demonstrated that there are two important types of MDE - *membrane-bound* and *diffusible* metalloproteinases - involved in cancer invasion and their study focussed on the membrane-bound membrane-type-1 matrix metalloproteinase (MT1-MMP) and the diffusible (soluble) matrix metalloproteinase-2 (MMP-2), and their interactions with the extracellular matrix.

Following the work in Deakin and Chaplain (2013), which was based on the experimental results in Sabeh et al. (2009), in this paper we consider models for cancer cell invasion mediated by enzyme based degradation of the ECM. Specifically our focus is on ECM degradation via both soluble and membrane-bound matrix metalloproteinases (MMPs). We propose a microscopic model which accounts for matrix degradation via these two pathways and formulate an effective macroscopic model valid in the limit where the size of the tissue is large relative to the size of the individual cells. Assuming a periodic or locally periodic distribution of cancer cells, the rigorous derivation of the macroscopic model can be obtained using homogenization methods namely, two-scale convergence and the unfolding operator, see e.g. Allaire (1992); Cioranescu et al. (2018); Nguetseng (1989); Ptashnyk (2013, 2015), using similar methods as developed in Marciniak-Czochra and Ptashnyk (2008); Ptashnyk and Venkataraman (2020). It is however extremely challenging, and beyond the scope of this work, to incorporate tumour invasion via movement and proliferation of the cancer cells in the microscopic model such that effective macroscopic equations can be rigorously derived from the microscopic description of the processes. We remark that a major obstacle in this direction is the lack of a sufficient theory for continuum modelling of the dynamics of cellular processes during proliferation. We instead propose a phenomenological approach to derive an effective macroscopic model for invasion in which microscopic features, specifically degradation of the ECM by membrane-bound MMPs, can be accounted for. Furthermore, we account for the influence of the microstructure on the transport of the soluble MMPs through the ECM by deriving an effective diffusion coefficient using homogenization methods. We note that the framework and methodology we propose for computing the effective diffusivity is applicable generally to multiphase models of tumour growth such as those mentioned previously, as well as other applications involving heterogeneous mixtures. Our numerical results demonstrate qualitative differences between the computed effective diffusion tensors in two versus three dimensions. We show existence of nonnegative and bounded solutions of the macroscopic model using a fixed-point argument together with the Galerkin method and the method of positive invariant regions. The model consists of a coupled system of ordinary differential equations and an anisotropic degenerate parabolic equation. The variable doubling method can be applied to show well-posedness for anisotropic degenerate parabolic equations with diffusion coefficients in $W^{1,\infty }(\Omega )$, see e.g. Chen and Perthame (2003); Chen and Karlsen (2005). However, in our case the dependence of the diffusion coefficient on the ECM density, which satisfies an ordinary differential equation, does not imply the required regularity. To prove the well-posedness results we use the fact that for strictly positive initial ECM density and for any finite time interval the ECM density is uniformly bounded from below by a positive constant. Numerical simulations of the macroscopic model illustrate the crucial role bound MMPs appear to play in cancer invasion, in agreement with previous results (Deakin and Chaplain 2013; Sabeh et al. 2009), as well as the fact that the action of bound MMPs can lead to spatial heterogeneity in the invasive front whilst the action of the soluble MMPs seems to generate more radially symmetric invasive profiles.

In summary, the key results of the work are

- Development of a framework in which detailed cell-scale models for ECM degradation can be incorporated in a macroscopic model for cancer cell invasion of the ECM that can be simulated at tissue scales.
- A methodology for computing the effective diffusivity of soluble molecules in heterogeneous medium representing cancer cells and ECM with varying volume fractions.
- The analysis of a coupled system of semilinear ODE-PDEs that serves as an effective model for cancer cell invasion of the ECM.
- Numerical simulation results of invasive processes illustrating qualitative features of the modelling such as the crucial role bound MMPs appear to play in determining both the speed of invasion and spatial heterogeneity in the invasive front.

The paper is organised as follows. In section 2 we formulate a microscopic model for ECM degradation through the action of bound and soluble MMPs which consists of a system of coupled bulk-surface equations. In section 3 we propose effective macroscopic models for cancer cell invasion of the ECM. In section 4 we prove the well posedness of the macroscopic model, i.e., the coupled semilinear ODE-PDE system. In section 5 we formulate a finite element scheme for the approximation of the macroscopic model. In section 6 we discuss the parameterisation of the model based, where possible, on experimentally measured parameter values, present numerical simulation results for the effective diffusivity of soluble MMPs for different volume fractions of the ECM in $2\textrm{d}$ and $3\textrm{d}$, and report on the results of numerical simulations of the macroscopic model that illustrate various aspects of cancer invasion. Finally, in section 7 we discuss our results as well as potential directions for future work.

## Microscopic description of cancer cell and ECM interactions

**Fig. 1:**
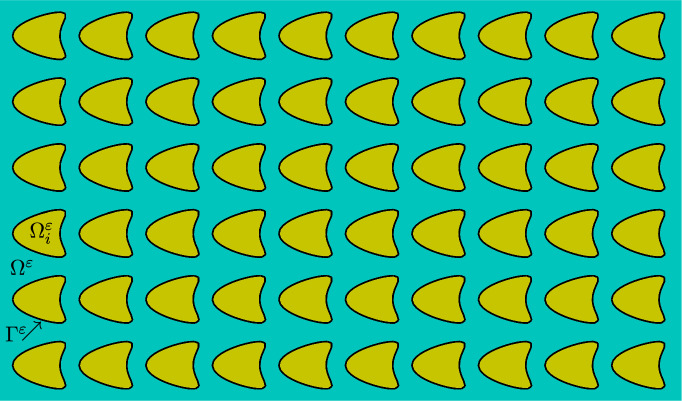
A sketch of the geometry consisting of cancer cells surrounded by the ECM. The domains $\Omega _i^{\varepsilon }, \Gamma ^\varepsilon$ and $\Omega ^\varepsilon$ corresponding to the cancer cells, cancer cell membranes and ECM are shaded yellow, black and blue respectively (color figure online)

To formulate the microscopic model, we consider processes at the level of single cells where ECM degradation occurs due to the action of soluble and membrane bound MMPs. We consider a convex Lipschitz, or $C^{1,1}$, domain $\Omega \subset \mathbb {R}^d$, with $d=2,3$, representing a part of a biological tissue and assume a time independent geometry and a periodic distribution of cells of the same shape. To describe the microscopic structure of the tissue, we consider a ‘reference domain’ $Y=(0,1)^d$, and the subdomains $\overline{Y}_i\subset Y$ and $Y_e = Y \setminus \overline{Y}_i$, together with the boundary $\Gamma = \partial Y_i$. The domain occupied by the cells is given by $\Omega _i ^\varepsilon = \bigcup _{\xi \in \Xi ^\varepsilon } \varepsilon (Y_i + \xi )$, where $\Xi ^\varepsilon = \{ \xi \in \mathbb Z^d, \; \; \varepsilon (\overline{Y}_i + \xi ) \subset \Omega \}$, the nonnegative parameter $\varepsilon$ represents the ratio between the diameter of a cancer cell and the tissue. The extracellular space is denoted by $\Omega ^\varepsilon = \Omega \setminus \overline{\Omega }_i^\varepsilon$. The surfaces that describe cell membranes are denoted by $\Gamma ^\varepsilon = \bigcup _{\xi \in \Xi ^\varepsilon } \varepsilon (\Gamma + \xi )$ and we assume they are smooth surfaces without boundary, see Figure 1 for a sketch of the geometry. As we are interested in tissues containing many cells we have $\varepsilon \ll 1$.

We let $c^\varepsilon _s:\Omega ^\varepsilon \times [0,T]\rightarrow {\mathbb {R}}$ denote the concentration of soluble MMPs that are produced by the cancer cells and diffuse in the ECM, $c_b^\varepsilon :\Gamma ^\varepsilon \times [0,T]\rightarrow {\mathbb {R}}$ denotes the surface density of bound MMPs on the cancer cell membranes and we let $m^\varepsilon : \Omega ^\varepsilon \times [0,T]\rightarrow {\mathbb {R}}$ and $m^\varepsilon _\Gamma : \Gamma ^\varepsilon \times [0,T]\rightarrow {\mathbb {R}}$ denote the ECM density in the extracellular domain and at the cell surface, respectivelly.

For $c_s^\varepsilon$ we propose the following dynamics

1 $$\begin{aligned} \begin{aligned}&\partial _t c_s^\varepsilon - \nabla \cdot (\bar{D}_s(x)\nabla c_s^\varepsilon ) = - g_s(c_s^\varepsilon , m^\varepsilon ) - \beta _s c_s^\varepsilon &  \text { in } (0,T] \times \Omega ^\varepsilon ,\\&\bar{D}_s(x)\nabla c_s^\varepsilon \cdot \nu = \varepsilon \kappa _s {f_s(c_s^\varepsilon , m^\varepsilon _\Gamma ) } &  \text { on } (0,T] \times \Gamma ^\varepsilon , \\&\bar{D}_s(x)\nabla c_s^\varepsilon \cdot \nu = 0 &  \text { on } (0,T] \times \partial \Omega , \\&c_s^\varepsilon (0) = c_{s, \mathrm in} &  \text { in } \Omega ^\varepsilon , \end{aligned} \end{aligned}$$

where $\nu$ is the normal pointing outwards of $\Omega ^\varepsilon$, $\bar{D}_s$ corresponds to the diffusivity of soluble MMPs in the ECM, $\kappa _s f_s(c_s^\varepsilon , m_\Gamma ^\varepsilon )$ describes the production and/or degradation of soluble enzymes by cells, $g_s(c_s^\varepsilon , m^\varepsilon )$ models the use of enzymes in degradation of ECM process and $\beta _s$ is the natural decay rate. Although, for simplicity, we consider $\bar{D}_s$ to depend only on the space variable, it could depend on the ECM density to account for effects due to changes in the pore size of the ECM. For $c_b^\varepsilon$ we consider

2 $$\begin{aligned} \begin{aligned}&\partial _t c_b^\varepsilon - \varepsilon ^2 \nabla _\Gamma \cdot ( D_b \nabla _\Gamma c_b^\varepsilon ) = \kappa _b { f_b(c_b^\varepsilon , m^\varepsilon _\Gamma ) }- g_b(c_b^\varepsilon , m^\varepsilon _\Gamma ) - \beta _b c_b^\varepsilon &  \text { on } (0,T]\times \Gamma ^\varepsilon ,\\&c_b^\varepsilon (0) = c_{b, \mathrm in}^\varepsilon &  \text { on } \Gamma ^\varepsilon , \end{aligned} \end{aligned}$$

where $D_b$ is the surface diffusivity of $c_b^\varepsilon$, $\kappa _b f_b(c_b^\varepsilon , m^\varepsilon _\Gamma )$ describes production of bound enzymes through activation of the corresponding receptors due to interactions between cancer cells and ECM, $\beta _b$ is the natural decay rate, $g_b(c_b^\varepsilon , m^\varepsilon _\Gamma )$ corresponds to the use of the cancer cell-bound enzymes in the degradation of ECM, and $c_{b, \mathrm in}^\varepsilon (x) = c_{b, \mathrm in}(x/\varepsilon )$ for $c_{b, \mathrm in}\in L^2(\Gamma )$, extended *Y*-periodically to $\mathbb R^d$. Finally for the ECM densities $m^\varepsilon$ and $m^\varepsilon _\Gamma$ we consider the following equations modelling the degradation of EMC due to interactions of bound and free MMPs with the ECM

3 $$\begin{aligned} \begin{aligned}&\partial _t m^\varepsilon = - g_s(c_s^\varepsilon , m^\varepsilon ) &  \text { in } (0,T]\times \Omega ^\varepsilon , \\&{\partial _t m_\Gamma ^\varepsilon = - g_b(c_b^\varepsilon , m^\varepsilon _\Gamma ) } &  \text { on } (0,T]\times \Gamma ^\varepsilon , \\&m^\varepsilon (0) = m_\textrm{in} \; \text { in } \Omega ^\varepsilon , \quad { m^\varepsilon _\Gamma (0) = m_{\Gamma ,\mathrm in}^\varepsilon } &  \text { on } \Gamma ^\varepsilon , \end{aligned} \end{aligned}$$

where $m_{\Gamma ,\mathrm in}^\varepsilon (x) = m_{\Gamma ,\mathrm in}(x/\varepsilon )$ for $m_{\Gamma ,\mathrm in} \in L^2(\Gamma )$, extended *Y*-periodically to $\mathbb R^d$. The $\varepsilon$-scalings in equations (1) and (2), ensuring a nontrivial limiting model when $\varepsilon \rightarrow 0$, are obtained through nondimensionalisation of the microscopic model. In this regard, we briefly discuss biologically relevant values for the various parameters, which suggest this scaling regime is reasonable, cf., also (Ptashnyk and Venkataraman 2020). Considering a representative cancer cell diameter of $10 \mu \textrm{m}$, we obtain that the parameter $\varepsilon$ representing the ratio between the size of a cell and the size of the tissue is of order $10^{-2}$ or smaller. In the microscopic model (1)-(3), the dimension for the parameter $\kappa _s$ in addition to the time scale will also depend on the representative size of cancer cells, resulting in the $\varepsilon$-scaling in the boundary condition on $\Gamma ^\varepsilon$ in the non-dimensionalised problem (1). For the equation on the cell membrane, the diffusion coefficient $D_b$ will be of order $10^{-11}-10^{-12} \textrm{cm}^2 \textrm{s}^{-1}$, see e.g. Linderman and Laufenberger (1986); Sako and Kusumi (1994); Simson et al. (1998); Valdez-Taubas and Pelham (2003), which naturally yields the $\varepsilon ^2$-scaling in the non-dimensional equation in (2) and hence dependence of $c_b$ on the microscopic cell-scale variable in the macroscopic model. Fast diffusion on the cell membrane, corresponding to a $\varepsilon$- or $\varepsilon ^0$- scaling in the diffusion term of the non-dimensional equation for $c_b^\varepsilon$, would result in the homogeneous distribution of membrane-bound MMPs on the cell membrane in the macroscopic tissue level model.

We note that dynamics of species in the cell interior that influence the production of MMPs can also be included in the microscopic model and the corresponding modelling and homogenization results are discussed in Ptashnyk and Venkataraman (2020).

For locally-Lipschitz continuous functions $f_s$, $f_b$, $g_b$, and $g_s$, such that

4 $$\begin{aligned} \begin{aligned}&|f_b(\eta , \xi )| + |f_s(\eta , \xi )| \le C(1+ |\eta |+ |\xi |) \; \text{ for } \eta , \xi \in \mathbb R, \, C>0, \; \\ &f_b(0, \xi )\ge 0,\,\; f_s(0, \xi ) \ge 0 \; \text{ for } \xi \ge 0, \\ &0 \le g_b(\xi , \eta ) \le C_b \xi \eta , \; \; 0 \le g_s(\zeta , \eta ) \le C_s\zeta \eta \; \; \text{ for } \; \xi , \eta , \zeta \ge 0 \; \text{ and } \; C_s>0, C_b >0, \\ &g_b(\xi , \eta ) =0 , \; \; g_s(\xi , \eta ) = 0 \; \text{ if } \; \xi =0 \; \text{ or } \; \eta =0, \end{aligned} \nonumber \\ \end{aligned}$$

and

5 $$\begin{aligned} \begin{aligned}&0< d_s \le \bar{D}_s(x) \le \hat{d}_s < \infty , \; \text{ for } x \in \Omega , \quad D_b, \, \beta _s, \, \beta _b, \, \kappa _b, \, \kappa _s >0, \\ &c_{s, \mathrm in}, \; m_\text {in} \in L^\infty (\Omega ), \quad c_{b, \mathrm in}, \; m_{\Gamma , \text {in}} \in L^\infty (\Gamma ), \\ &\text{ with } \; c_{s, \mathrm in}\ge 0, \; m_\text {in} \ge 0 \; \text{ in } \Omega , \quad c_{b, \mathrm in} \ge 0, \; m_{\Gamma , \text {in}} \ge 0 \; \text{ on } \Gamma , \end{aligned} \end{aligned}$$

using homogenization methods, in the same way as in Marciniak-Czochra and Ptashnyk (2008); Ptashnyk and Venkataraman (2020), one can derive the corresponding tissue-level (macroscopic) two-scale model

6 $$\begin{aligned} \partial _t c_s - \nabla \cdot ( D_{s}^\text {hom}(x) \nabla c_s)&= \kappa _s \frac{1}{ |Y_e|} \int _\Gamma f_s(c_s, m_\Gamma ) d\gamma \nonumber \\ &\qquad - g_s (c_s, m) - \beta _s c_s&\text{ in } (0,T] \times \Omega ,\nonumber \\ \partial _t c_b - \nabla _{\Gamma , y}\cdot (D_b \nabla _{\Gamma , y} c_b)&= \kappa _b f_b(c_b, m_\Gamma ) \nonumber \\ &\qquad - g_b(c_b, m_\Gamma ) - \beta _b c_b&\text{ in } (0,T] \times \Omega \times \Gamma ,\nonumber \\ \partial _t m&= - g_s(c_s, m)&\text{ in } (0,T] \times \Omega ,\nonumber \\ \partial _t m_\Gamma&= - g_b(c_b,m_\Gamma )&\text{ in } (0,T] \times \Omega \times \Gamma ,\nonumber \\ D_{s}^\text {hom} (x)\nabla c_s \cdot \nu&= 0&\text{ on } (0,T] \times \partial \Omega ,\nonumber \\ c_s(0) = c_{s, \mathrm in}, \qquad&m(0) = m_\text {in}&\text{ in } \; \Omega , \quad \; \nonumber \\ c_b(0) =c_{b, \mathrm in}, \qquad&m_\Gamma (0) = m_{\Gamma , \text {in}} \;&\text { in } \; \Omega \times \Gamma . \end{aligned}$$

The effective diffusion matrix is given by

7 $$\begin{aligned} D^\textrm{hom}_{s,ij}(x)= \frac{1}{|Y_e|} \int _{Y_e} \big [ \bar{D}_{s}(x) \delta _{ij} + \big (\bar{D}_{s}(x) \nabla _y w^j(y)\big )_i \big ] dy, \quad \text { for }\;\; i,j=1,\dots ,d, \end{aligned}$$

with $w^j$ being *Y*-periodic solutions of the unit cell problems

8 $$\begin{aligned} \begin{aligned}&\nabla _y\cdot \big ( \bar{D}_s(x)(\nabla _y w^j + e_j)\big ) = 0 &  \text { in } Y_e \times {\Omega }, \quad \int _{Y_e} w^j(x,y) dy = 0, \\&\bar{D}_s(x)(\nabla _y w^j + e_j) \cdot \nu = 0 &  \text { on } \Gamma \times {\Omega }, \quad w^j(x, \cdot ) \; \; Y-\text {periodic}, \end{aligned} \end{aligned}$$

for $x\in \Omega$ and $j=1, \ldots , d$, where $\{ e_j\}_{j=1, \ldots , d}$ is the standard basis in $\mathbb R^d$. The above assumptions on the nonlinear functions $f_s, f_b, g_s, g_b$ also ensure the well-posedness of the microscopic (1)-(3) and macroscopic (6) models, see (Marciniak-Czochra and Ptashnyk 2008; Ptashnyk and Venkataraman 2020).

The model (1)-(3) and the corresponding macroscopic model (6) describe cancer cell-ECM dynamics but not invasion processes, as in the latter case we must account for movement and proliferation of cancer cells. For proliferation, one would have to include topological changes for which there is as yet no rigorous theory and hence we do not account for this even at the microscopic level. Movement of the cancer cells could be incorporated by deriving the corresponding equations on time dependent domains, this has been the subject of a number of recent works, e.g., MacDonald et al. (2016); Alphonse et al. (2018, 2024). However our goal is to understand tissue level models in the limit $\varepsilon \rightarrow 0$ and to do this we assume separation of scales for processes happening at the cell and tissue level respectively. Thus we do not include movement in the microscopic models described above and instead in section 3 we propose a framework that phenomenologically accounts for movement and proliferation of cancer cells at the tissue level inspired by the above microscopic description of processes occurring at the cell level. A rigorous homogenisation of a microscopic model that includes cell movement is an interesting topic which we intend to consider in a future study.

## Tissue level modelling of MMP mediated cancer cell invasion

In this section we propose a macroscopic model for cancer cell invasion into the ECM. We combine aspects of mixture and multiscale modelling approaches to model the effect of bound and soluble MMPs on ECM degradation and cancer invasion. We denote the volume fraction of the ECM by $\phi (t,x)$ and make the modelling assumption that the corresponding volume fraction of tumour cells is given by $\psi (t,x) = 1- \phi (t,x)$ for $x\in \Omega$ and $t\in [0,T]$. In effect, we are assuming that as the cancer cells degrade the ECM they instantaneously occupy the resultant space, via proliferation and movement, both of which are not explicitly modelled. The modelling formalism we employ essentially consists of a mixture model or two-phase model with the tissue consisting of a mixture of cells and ECM such that their combined volume fraction is always equal to one. Such a modelling formalism is widely used in models for tumour or tissue growth, see e.g., Byrne and Preziosi (2003) and in our case serves as a phenomenological framework for modelling invasion in the present study. Accounting for movement and proliferation of cells is an extremely interesting direction, especially in the latter case as topological changes must be considered in the microscopic model. We intend to focus on this in future work. We present two modelling frameworks for investigating MMP mediated invasion that take into account the cell-scale features discussed in section 2. The macroscopic model described in section 3.1 does not include heterogeneity of the dynamics at the cell level but takes into account changes in the tissue microstructure due to cancer invasion, whilst the two-scale model, outlined in section 3.2 is similar in structure to the models rigorously derived in Ptashnyk and Venkataraman (2020) and allows spatial heterogeneity in the intracellular processes inherent in the model of section 2 to be incorporated.

### Macroscopic model

In formulation of a macroscopic model we assume that the MMPs can be described by concentrations at the tissue scale, i.e., we assume spatial homogeneity at the cell level. As in section 2, the concentration of soluble matrix degrading enzymes is denoted by $c_s(t,x)$ and the whilst the surface density of membrane bound matrix degrading enzymes is now given by $c_b(t,x)(1-\phi (t,x))$ for $x\in \Omega$ and $t\in [0,T]$. We note the dependence on the volume fraction of cancer cells in the latter expression as the membrane bound species are bound to the membranes of the cancer cells. The evolution equations for the ECM and MMPs we propose are as follows

9 $$\begin{aligned} \left. \begin{aligned}&\partial _t \phi = - \mu _b \phi \, c_b(1-\phi ) - \mu _s \phi c_s\\&\partial _tc_s - \nabla \cdot (D_s(\phi ) \nabla c_s)\\&\quad = \kappa _s f_s(c_s)(1- \phi ) - \mu _s \phi c_s - \beta _s c_s \\&\partial _t (c_b(1- \phi )) \\&\quad = \kappa _b f_b(\phi )(1- \phi ) - \mu _b \phi c_b(1-\phi ) - \beta _b c_b (1- \phi ) \end{aligned} \right\} \text { in }{\Omega }\times (0,T], \end{aligned}$$

where we have accounted for degradation of the ECM by bound and soluble MMPs, ECM volume fraction dependent diffusion of the soluble MMPs in the tissue, see section 3.3 for details on how this is modelled, production of soluble and bound MMPs by the cancer cells, degradation of the ECM by the soluble and insoluble MMPs and the corresponding use of MMPs, at rates $\mu _s$ and $\mu _b$ respectively, and linear decay of the MMPs. For the degradation of the ECM by the MMPs we have made the simplest relevant assumption that this is linear with respect to the concentration of the MMPs. For the reaction terms governing the production of MMPs by the cancer cells we assume a Hill function form Martinez-Corral et al. (2024), i.e.,

10 $$\begin{aligned} f_b(\phi ) = \frac{\phi }{1+\phi }, \quad f_s(c_s) = \frac{1}{ 1+ c_s}. \end{aligned}$$

More general models where $f_b$ depends also on $c_b(1-\phi )$ and $f_s$ on $\phi$, as we allow in the microscopic model (1) and (2), can be included without major complications, given suitable growth conditions. To close the model, we consider zero flux boundary condition for $c_s$ and the initial conditions

11 $$\begin{aligned} \begin{aligned}&D_s(\phi ) \nabla c_s\cdot \nu = 0 \; \; &  \text { on } \partial \Omega \times (0,T], \\&\phi (0,x) = \phi _0(x), \; \; \; c_s(0,x) = c_{s,0}(x), \; \; c_b(0,x) = c_{b,0}(x) \; \; &  \text { for } x\in \Omega . \end{aligned} \end{aligned}$$

For biological relevance, we assume $\phi _0, c_{s,0}$ and $c_{b,0}$ to be bounded nonnegative functions,

12 $$\begin{aligned} \begin{aligned}&0 < \delta _0\le \phi _0(x)\le 1, \quad 0 \le c_{s,0}(x)\le M_{s,0}, \quad 0 \le c_{b,0}(x) \le M_{b,0} \; \;\text { for } \; x \in \Omega , \\&c_{s,0} \in H^1(\Omega ), \quad \phi _0, \, c_{b,0}(1-\phi _0) \in W^{1,p}(\Omega ) \text { for } p\ge 4, \\&{ D_s \in \textrm{Lip}_\textrm{loc}(\mathbb R)^{d\times d}, \; D_s(\xi ) \eta \eta \ge d(\xi ) |\eta |^2 \text { for } \eta \in \mathbb R^d, \text { with }}\\&d(\xi ) \ge d_s \xi \text { for } d_s >0, \text { and } \xi \in \mathbb R_+. \end{aligned} \end{aligned}$$

The assumption that the initial condition for $\phi$ is strictly positive is needed in our proof of the well-posedness of model (9), (11). The strict positivity of $\phi _0$ will imply the strict positivity of solutions $\phi$ of model (9), which in turn ensures that $D_s(\phi )$ is uniformly positive definite and a priori estimates for $\nabla c_s$. The uniform estimates for $\nabla c_s$ are essential for the proof of the well-posedness results, in particular for the proof of strong convergence used in the fixed-point argument. Removing the restriction of strict positivity of initial conditions for $\phi$ leads to a degenerate nonlinear parabolic equation for $c_s$. There are some results for degenerate nonlinear parabolic equations, which rely on the specific structure of the singularity, that allow one to prove well-posedness results for kinetic or entropy solutions in $L^1$, see e.g. Chen and Perthame (2003); Chen and Karlsen (2005). However similar ideas can not be applied to problem (9), (11) due to strong coupling between solutions of the degenerate parabolic problem and solutions of the ordinary differential equations and the dependence of the degeneracy in the elliptic operator on the solutions of the ordinary differential equations. Therefore development of a new approach for systems of such type is needed, this is an interesting topic for future work. The $H^1$- and $W^{1,p}$-regularity of initial conditions is required only for the proof of uniqueness of solutions.

### Two-scale model

If cell-scale heterogeneity in the spatial distribution of membrane bound MMPs is relevant, this is in effect governed by the details of the microscopic model, then a two-scale model of the following form may be considered. In the absence of invasion this model can be derived from the microscopic model in section 2 cf., Ptashnyk and Venkataraman (2020). For the two-scale model we let $c_b(t,x,y)(1-\phi (t,x))$ for $y\in \Gamma , x\in \Omega$ and $t\in [0,T]$ denote the density of membrane bound MMPs at a point *x* in the tissue, *y* on the membrane and time *t*. The corresponding system of equations reads13Comparing with (9), system (13) includes the dynamics of a spatially heterogenous distribution of $c_b$ on the cell membrane. For simplicity, in the description above we have assumed that all cells are of the same shape and size. Using techniques from locally-periodic homogenization, see e.g. Ptashnyk (2015), we can also derive the above system if cells varied in shape with respect to *x* and *t*, e.g. $\Gamma _{t,x}= \vec {\mathcal {A}}(t,x) \Gamma$, with some given diffeomorphism $\vec {\mathcal {A}}$, where $\Gamma$ is a reference membrane. Using this approach we could incorporate the deformation and/or growth of cells, c.f., Lakkis et al. (2024) for an example of such a two-scale model and its numerical resolution.

### Effective diffusion tensor

**Fig. 2:**
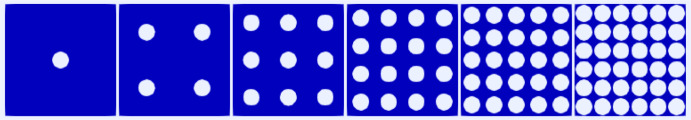
Domains used for computing the effective diffusion tensor in $2\textrm{d}$ corresponding to volume fractions of ECM of 0.982, 0.929, 0.841, 0.717, 0.558 and 0.364 reading from left to right

**Fig. 3:**
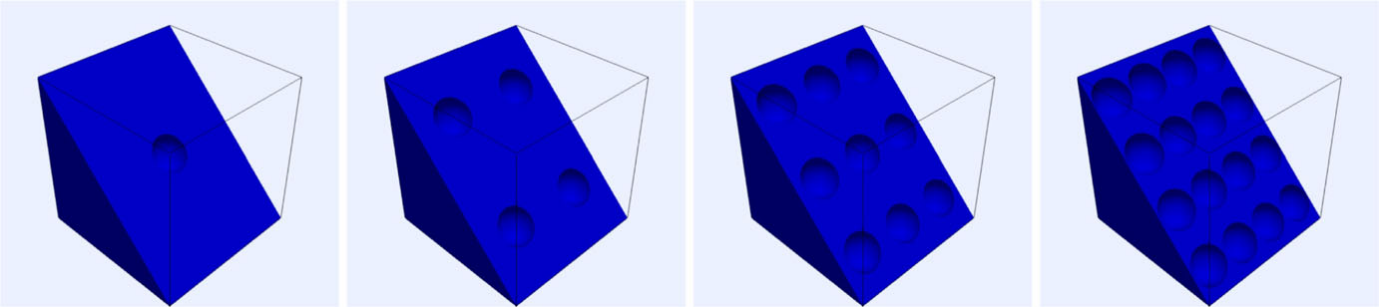
Domains used for computing the effective diffusion tensor in $3\textrm{d}$ corresponding to volume fractions of ECM of 0.993, 0.942, 0.805 and 0.537 reading from left to right. Half of the domain has been made transparent in each case to visualise the interior boundaries

**Fig. 4:**
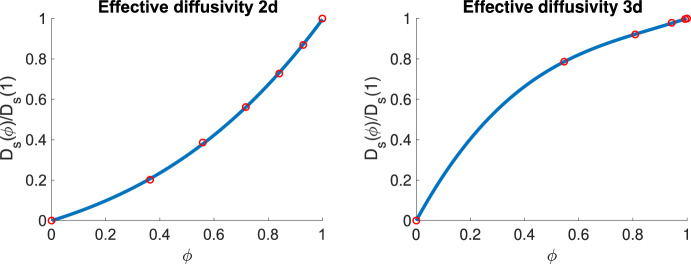
Fitted effective diffusivity in $2\textrm{d}$ and $3\textrm{d}$. The red circles correspond to computed effective diffusivities obtained by solving (8) on domains described in section 6.2. The blue curves are least squares cubic polynomial fits using these values (Color figure online)

Both the single-scale and two-scale macroscopic models contain an effective diffusion coefficient that describes the diffusivity of the soluble MMPs in the tissue. This diffusivity is a nonlinear function of $\phi$, the volume fraction of the extracellular matrix. For the reference domain *Y*, the corresponding value of $\phi$ is obtained by computing $\vert Y_e\vert$ and the effective diffusion matrix is computed as in (7) and (8). To obtain the function $D_s(\phi )$ we compute $D^\textrm{hom}_{s}$ for a number of different values of $\phi$ i.e., on different reference domains, and fit an effective diffusivity on these values, see section 6.2 and Appendix A as well as Figures 2, 3 and 4. We note that the fitted effective diffusivity should satisfy that $D_s(\phi )$ is positive definite for all $\phi \in (0,1]$. In Appendix 7.1 we remark on one approach towards ensuring this for the general case of diffusion tensors which are not diagonal.

###  Macroscopic model with heterogeneous extracellular matrix

Following (Deakin and Chaplain 2013), we introduce a matrix suitability factor $s:\Omega \times [0,T]\rightarrow [0,1]$, which models heterogeneity in the ECM due, for example, to variations in pore size or fibre density. We assume the ability of cancer cells to invade the ECM is a decreasing function of *s*, i.e., $s=1$ and $s=0$ correspond to ECM configurations that are the least and most suitable for cancer cell invasion, respectively. We further assume that only the membrane bound MMPs can change the suitability of the ECM for invasion (Sabeh et al. 2009). Assuming a simple linear dependence of the invasion related parameters on *s* and that the only effect of matrix suitability is to inhibit the degradation of matrix by MMPs yields the model:

14 $$\begin{aligned} \left. \begin{aligned} \partial _t \phi&= - (1-s)\mu _b \phi \, c_b(1-\phi ) - (1-s)\mu _s \phi c_s\\ \partial _tc_s -\nabla \cdot (D_s(\phi ) \nabla c_s)&= \kappa _s f_s(c_s)(1- \phi ) \\ &\quad - (1-s)\mu _s \phi c_s - \beta _s c_s \\ \partial _t (c_b(1- \phi ))&= \kappa _b f_b(\phi )(1- \phi ) \\ &\quad - (1-s)\mu _b \phi \, c_b(1-\phi ) - \beta _b c_b (1- \phi )\\ \partial _t s&= - \delta _s c_b(1-\phi )s \end{aligned} \right\} \text { in }{\Omega }\times (0,T], \end{aligned}$$

with boundary and initial conditions as in (11) and additional initial condition $s(0, x) = s_0(x)$, where $0 \le s_0(x) \le 1$ for $x \in \Omega$. One could also consider that the diffusion coefficient $D_s$ depends on *s* but we do not include this in the current work to allow for comparison with the results in Deakin and Chaplain (2013).

## Analysis of the macroscopic models

Following the same arguments as in Ptashnyk and Venkataraman (2020) we can prove the following well-posedness result for the macroscopic two-scale model (6).

### Theorem 4.1

Under assumptions (4), (5) there exists a unique weak solution $c_s \in L^2(0,T; H^1(\Omega ))$, $c_b \in L^2((0,T)\times \Omega ; H^1(\Gamma ))$, $m \in H^1(0,T; L^2(\Omega ))$, $m_\Gamma \in H^1(0,T; L^2(\Omega \times \Gamma ))$ of the two-scale problem (6), with $c_s(t,x), c_b(t,x,y), m(t,x), m_\Gamma (t,x,y) \ge 0$ for $(t,x) \in (0,T)\times \Omega$ and $y \in \Gamma$, $c_s, m \in L^\infty ((0,T)\times \Omega )$, $c_b, m_\Gamma \in L^\infty ((0,T)\times \Omega \times \Gamma )$, and $\partial _t c_s \in L^2(0,T; H^1(\Omega )^\prime )$, $\partial _t c_b \in L^2((0,T)\times \Omega ; H^1(\Gamma )^\prime )$.

The challenges in proving the existence and uniqueness of solutions to the macroscopic model (9), (11) arise due to the fact that the system consists of coupled ODEs and PDEs with a possibly degenerate diffusion. For the model with heterogeneous suitability (14) the proof of the well-posedness is similar to that for (9), (11) and hence omitted.

A weak solution of system (9) with initial and boundary conditions (11) is defined as follows.

### Definition 4.2

Functions $\phi , c_b(1-\phi ) \in H^1(0,T; L^2(\Omega )) \cap L^\infty ((0,T)\times \Omega )$ and $c_s \in L^\infty (0,T; L^2(\Omega )) \cap L^2(0,T; H^1(\Omega ))$ are weak solutions of (9), (11) if

15 $$\begin{aligned}&\int _0^T\!\!\Big [ -\langle c_s, \partial _t w \rangle + \langle D_s(\phi ) \nabla c_s, \nabla w \rangle \Big ] dt\nonumber \\&\quad =\int _0^T \!\!\langle \kappa _s f_s(c_s) (1-\phi ) - \mu _s \phi c_s - \beta _s c_s, w \rangle dt + \langle c_{s, 0} , w(0) \rangle \end{aligned}$$

for every $w \in L^2(0,T; H^1(\Omega ))$, with $\partial _t w \in L^2((0,T)\times \Omega )$ and $w(T) = 0$, and the equations and initial conditions for $\phi$ and $c_b(1-\phi )$ are satisfied a.e. in $(0,T)\times \Omega$.

Here we introduced the notation $\langle u, v \rangle = \int _\Omega u v dx$, for $u\in L^p(\Omega )$ and $v \in L^q(\Omega )$ with $1/p+1/q =1$.

### Theorem 4.3

Under the assumptions (12) there exists a unique weak solution of problem (9),(11), satisfying

$$ 0\le \phi (t,x) \le 1, \quad 0 \le c_b(t,x)(1- \phi (t,x)) \le M_b, \quad 0 \le c_s(t,x) \le M_s , $$

for $x \in \Omega$ and $t\ge 0$, where $M_b= \max \{M_{b,0}, {\kappa _b}/{\beta _b}\}$ and $M_s= \max \{M_{s,0}, {\kappa _s}/{\beta _s}\}$.

Additionally over any finite time interval [0, *T*] we have

16 $$\begin{aligned} \phi (t,x) \ge \tilde{\delta }= \delta _0 \exp (- (\mu _b \kappa _b/\beta _b + \mu _s \kappa _s/\beta _s) T) >0. \end{aligned}$$

### Remark 4.1

The proof of Theorem 4.3 is structured as follows.

- First we use a fixed point argument considering a given $0< \tilde{\delta }\le \bar{\phi }\le 1$ in the equation for $c_s$, see (17), which decouples it from the equations for $\phi$ and $c_b$ and ensures uniform ellipticity.
- Applying the method of positive invariant regions for ODEs, e.g. Amann (1990), and for reaction-diffusion equations, e.g. Smoller (1994), we show nonegativity and boundedness of solutions of (17), (11), assuming their existence. The same arguments ensure the nonegativity and boundedness for the Galekin approximations, and hence also for the limits of the approximation sequences. Note that convex sets are weakly sequentially closed.
- Then using the Galerkin method we prove the existence of a weak solution of the problem with a given $\bar{\phi }$ in the equation for $c_s$, see (17).
- To pass to the limit in the nonlinear terms we prove the strong convergence of the Galerkin approximation by showing the Cauchy properties of the corresponding sequences.
- To complete the existence proof we show that the map $\bar{\phi }\mapsto \phi$, defined by solutions of problem (17), (11), has a fixed point by proving the strong convergence of the corresponding sequences and applying the Schauder fixed-point theorem. To show strong convergence in $L^2((0,T)\times \Omega )$ we combine the Cauchy properties for solutions of the ODEs with the regularity properties and Aubin-Lions compactness lemma for solutions of the reaction-diffusion equation.
- Local Lipschitz continuity of the nonlinear functions and boundedness of solutions are applied to prove uniqueness.
- To estimate the terms arising due to the diffusion matrix $D_s$ being a function of $\phi$ we use higher regularity properties of solutions of the parabolic equations and the structure of the ODEs, together with the fact that the regularity of $\phi$ and $c_b(1-\phi )$ with respect to the space variables is determined by the regularity of $c_s$.

### Proof

To prove the existence result we combine the fixed point and Galerkin methods. For given

$$\begin{aligned} \bar{\phi }\in V, \; \; \text { where } \; V =\big \{ v \in L^2((0,T)\times \Omega ) \, : \; 0 < \tilde{\delta }\le v(t,x) \le 1 \; \text { for } \; (t,x) \in (0,T)\times \Omega \big \}, \end{aligned}$$

consider the map $K: V \rightarrow V$ with $\phi = K (\bar{\phi })$ being the solution of system

17 $$\begin{aligned} \begin{aligned} \partial _t \phi&= - \mu _b \phi c_b (1- \phi ) - \mu _s \phi c_s, \\ \partial _t (c_b(1- \phi ))&= \kappa _b f_b(\phi )(1- \phi ) - \mu _b \phi c_b(1-\phi ) - \beta _b c_b (1- \phi ),\\ \partial _tc_s - \nabla \cdot (D_s(\bar{\phi }) \nabla c_s)&= \kappa _s f_s(c_s)(1- \bar{\phi }) - \mu _s \bar{\phi }c_s - \beta _s c_s, \end{aligned} \end{aligned}$$

with initial and boundary conditions as in (11).

For $\tilde{\delta }\le \bar{\phi }\le 1$, applying the method of positive invariant regions, see e.g. Smoller (1994) and using the nonnegativity of $c_{s,0}$ we obtain $c_s (t,x)\ge 0$ for $x \in \Omega$ and $t\ge 0$. Using the theory of invariant regions for ordinary differential equations (ODEs), see e.g. Amann (1990) and $\phi _0(x)\ge 0$ for $x \in \Omega$, from the equation for $\phi$ it directly follows that $\phi (t,x) \ge 0$ for $x \in \Omega$ and $t\ge 0$. Considering first the positive part $(1-\phi )_{+}$ instead of $(1-\phi )$ in the first term on the right hand side of the equation for $c_b$ and using the assumptions on $c_{b,0}$ and $\phi _0$, we obtain $c_b(t,x)(1- \phi (t,x)) \ge 0$ for $x \in \Omega$ and $t\ge 0$. The non-negativity of $c_s$ and $c_b(1-\phi )$ and the fact that $\phi _0(x) \le 1$ imply that $\phi (t,x) \le 1$ for $x \in \Omega$ and $t\ge 0$. Then the assumption $\phi _0(x) \ge \delta _0$ ensures the lower bound (16) for any finite time interval [0, *T*]. Hence, the equations for $c_b$ with $(1-\phi )_+$ and with $(1-\phi )$ are equivalent. Considering the properties of the reaction terms and applying the method of positive invariant regions we also obtain $c_s\le M_s$ and $c_b(1- \phi ) \le M_b(1- \phi )$.

To show existence of solutions of (17) for $\bar{\phi }\in V$, we apply the Galerkin method and consider the sequence of approximations $\{\phi ^k\}$, $\{c_s^k\}$ and $\{c_b^k\}$ for solutions of system (17) given by

$$ \phi ^k(t,x) = \sum _{j=1}^k a_j^k(t) \psi _j(x), \quad c^k_b (t,x)= \sum _{j=1}^k b_j^k(t) \psi _j(x), \quad c^k_s (t,x)= \sum _{j=1}^k d_j^k(t) w_j(x), $$

and satisfying

18 $$\begin{aligned} \begin{aligned}&\langle \partial _t \phi ^k, \psi _j\rangle = - \langle \mu _b \phi ^k \, c_b^k(1-\phi ^k) + \mu _s \phi ^k c_s^k, \psi _j \rangle , \\&\langle \partial _t (c_b^k(1- \phi ^k)), \psi _j \rangle = \langle \kappa _b f_b(\phi ^k)(1- \phi ^k) - \mu _b \phi ^k c_b^k(1- \phi ^k) - \beta _b c_b^k (1- \phi ^k), \psi _j \rangle ,\\&\langle \partial _t c_s^k, w_j \rangle + \langle D_s( \bar{\phi }) \nabla c^k_s, \nabla w_j \rangle = \langle \kappa _s f_s(c_s^k)(1- \bar{\phi }) - \mu _s \bar{\phi }c^k_s - \beta _s c_s^k, w_j \rangle , \end{aligned} \end{aligned}$$

for $t \in (0,T]$ and initial conditions

19 $$\begin{aligned} \phi ^k(0,x) = \phi ^k_0(x), \quad c^k_b(0,x) = c^k_{b,0}(x), \quad c_s^k(0,x) = c^k_{s,0}(x), \end{aligned}$$

where $\{\psi _j\}_{j \in \mathbb N}$ are the basis functions of $L^2(\Omega )$ and $\{w_j\}_{j\in \mathbb N}$ are the basis functions of $H^1(\Omega )$, and $\phi ^k_0$, $c_{b,0}^k$ and $c_{s,0}^k$ are projections of $\phi$, $c_{b.0}$ and $c_{s,0}$ on $\text {span}\{\psi _1, \ldots , \psi _k\}$ and $\text {span}\{w_1, \ldots , w_k\}$ respectively. Notice that the same arguments as above ensure nonnegativity and boundedness for $\phi ^k, c_s^k$ and $c_s^k(1-\phi ^k)$. For given $\bar{\phi }$ the equation for $c_s^k$ in (18) is decoupled from the equations for $\phi ^k$ and $c_b^k$ and comprises a system of ODEs for $d_j^k$, with $j=1, \ldots , k$. Since $f_s$ is Lipschitz-continuous, ODE-theory, see e.g. Amann (1990), ensures existence of a unique solution $(d_1^k, \ldots , d_k^k)$ which is absolutely continuous. The local Lipschitz continuity of the nonlinear functions in the equations for $\phi ^k$ and $c_b^k$ and the boundedness of $\phi ^k$ and $c_b^k$ ensure the existence of a unique absolutely continuous solution of the systems of ODEs for $(a_1^k,\ldots , a_k^k, b_1^k, \ldots , b_k^k)$.

Taking $\phi ^k|\phi ^k|^{p-2}$, $c_b^k(1-\phi ^k)|c_b^k(1- \phi ^k)|^{p-2}$, for $p\ge 2$, and $c_s^k$ as test functions in equations for $\phi ^k$, $c_b^k$, and $c_s^k$ respectively, and using the nonnegativity and boundedness of $\phi ^k$, $c_b^k(1-\phi ^k)$ and $c_s^k$, we obtain the following a priori estimates

20 $$\begin{aligned} \begin{aligned}&\sup _{(0,T)} \Vert \phi ^k(t) \Vert ^p_{L^p(\Omega )} \le \Vert \phi (0) \Vert ^p_{L^p(\Omega )}, \quad \text { for } 1<p<\infty , \\&\sup _{(0,T)} \Vert c_b^k(t)(1-\phi ^k(t)) \Vert ^p_{L^p(\Omega )} \le C\big (1+ \Vert \phi (0) \Vert ^p_{L^p(\Omega )} \big ),\\&\sup _{ (0,T)} \Vert c_s^k(t) \Vert ^2_{L^2(\Omega )} + \Vert (d(\bar{\phi }))^{\frac{1}{2}} \nabla c_s^k \Vert ^2_{L^2((0,T)\times \Omega )} \le C,\\ \end{aligned} \end{aligned}$$

where the constant $C>0$ depends on *T*, $M_b$, and $M_s$ and is independent of *k*. Using (20), from the equations for $\phi ^k$, $c_b^k$, and $c_b^k$ we also obtain

21 $$\begin{aligned} \Vert \partial _t \phi _k \Vert _{L^2((0,T)\times \Omega )} + \Vert \partial _t (c_b^k(1-\phi _k)) \Vert _{L^2((0,T)\times \Omega )} + \Vert \partial _t c_s^k\Vert _{L^2(0,T; (H^1(\Omega )^\prime )}\le C, \end{aligned}$$

see e.g. Evans (2010) for the derivation of the estimate for $\partial _t c_s^k$. To pass to the limit in the nonlinear terms we require strong or almost everywhere convergence of $\{\phi ^k\}$, $\{c_s^k\}$ and $\{c_b^k\}$. To this end we show that $\{\phi ^k\}$, $\{c_s^k\}$ and $\{c_b^k(1- \phi ^k)\}$ are Cauchy sequences. The uniform boundedness of $\phi ^k$, $c_s^k$ and $c_b^k$ and the equations for $\phi ^k - \phi ^l$, $c_s^k - c^l_s$ and $c_b^k(1- \phi ^k) - c_b^l(1- \phi ^l)$ yield

22 $$\begin{aligned} \begin{aligned}&\Vert c_s^k (t)- c_s^l(t) \Vert _{L^2(\Omega )}^2 + \Vert (d(\bar{\phi }))^{\frac{1}{2}} \nabla (c_s^k - c_s^l) \Vert _{L^2((0,t)\times \Omega )}^2 + \Vert \phi ^k (t)- \phi ^l(t)\Vert _{L^2(\Omega )}^2 \\&\quad + \Vert c_b^k(t)(1- \phi ^k(t)) - c_b^l(t)(1-\phi ^l(t)) \Vert _{L^2(\Omega )}^2\le \Vert \phi ^k_0- \phi ^l_0\Vert _{L^2(\Omega )}^2 + \Vert c_{s,0}^k- c_{s,0}^l\Vert _{L^2(\Omega )}^2\\&\quad + \Vert c_{b,0}^k(1-\phi _0^k) - c_{b,0}^l(1-\phi ^l_0) \Vert _{L^2(\Omega )}^2 + C \!\int _0^t \!\Big [ \Vert \phi ^k(\tau )- \phi ^l(\tau )\Vert _{L^2(\Omega )}^2 \\&\quad + \Vert c_s^k (\tau )- c_s^l(\tau ) \Vert _{L^2(\Omega )}^2 + \Vert c_b^k(\tau )(1- \phi ^k(\tau ))- c_b^l(\tau )(1-\phi ^l(\tau ))\Vert _{L^2(\Omega )}^2 \Big ] d\tau . \end{aligned} \nonumber \\ \end{aligned}$$

Using the strong convergence in $L^2(\Omega )$ of the Galerkin approximations of the initial conditions, e.g. $\phi ^k_0 \rightarrow \phi _0$, $c_{s,0}^k \rightarrow c_{s,0}$, and $c_{b,0}^k \rightarrow c_{b,0}$ strongly in $L^2(\Omega )$ as $k \rightarrow \infty$, and applying the Gronwall inequality we obtain

23 $$\begin{aligned}&\sup _{(0,T)}\Vert \phi ^k- \phi ^l\Vert _{L^2(\Omega )}^2 + \sup _{(0,T)} \Vert c_s^k - c_s^l \Vert _{L^2(\Omega )}^2 + \sup _{(0,T)} \Vert c_b^k (1 - \phi ^k) - c_b^l(1-\phi ^l) \Vert _{L^2(\Omega )}^2\nonumber \\ \quad&\le \sigma (k,l), \end{aligned}$$

where $\sigma (k,l) \rightarrow 0$ as $k, l \rightarrow \infty$. The Cauchy property (23) implies the strong convergence of $\{\phi ^k\}$, $\{c_s^k\}$ and $\{c_b^k (1- \phi ^k)\}$ in $L^2((0,T)\times \Omega )$. The strong convergence of $\phi _k$ and $c_b^k (1- \phi ^k)$ ensures the convergence $c_b^k (1- \phi ^k) \rightarrow c_b (1- \phi )$ a.e. in $(0,T)\times \Omega$. Then the a priori estimates (20) and (21), uniform in *k*, together with convergence a.e. of $c_b^k (1- \phi ^k)$, imply

24 $$\begin{aligned} \begin{aligned}&c^k_s \rightharpoonup c_s \qquad \qquad \text {weakly-* in } L^\infty (0,T; L^2(\Omega )), \; &  \text { weakly in } L^2(0,T; H^1(\Omega )), \\&c_s^k \rightarrow c_s, \quad \phi ^k \rightarrow \phi , \quad c_b^k(1-\phi ^k) \rightarrow c_b(1- \phi ) \qquad &  \text { strongly in } L^2((0,T)\times \Omega ), \\&\partial _t c_s^k \rightharpoonup \partial _t c_s &  \text { weakly in } L^2(0,T; H^1(\Omega )^\prime ), \\&\partial _t \phi ^k \rightharpoonup \partial _t \phi , \quad \partial _t (c_b^k(1-\phi ^k)) \rightharpoonup \partial _t (c_b (1 -\phi )) \qquad &  \text { weakly in } L^2((0,T)\times \Omega ), \end{aligned} \end{aligned}$$

as $k \rightarrow \infty$. Using the convergence results in (24) we can pass to the limit as $k\rightarrow \infty$ in equations (18) and obtain that $\phi$, $c_s$, and $c_b$ are solutions of (17).

To prove the existence of a fixed point of the map *K*, we apply the Schauder fixed point theorem and show that $K: V \rightarrow V$ is compact, see e.g. Evans (2010). For this, by showing equicontinuity and, applying Riesz-Fréshet-Kolmogorov convergence result, see e.g. Brézis (2011), we prove strong convergence of a sequence of solutions $(\phi ^n, c_b^n, c_s^n)$ of problem (17), (11) with $\bar{\phi }^n \in V$. Using the a priori estimates for solutions of (17), (11), obtained from (20) and (21) by taking the limit as $k\rightarrow \infty$ and employing the lower semi-continuity of a norm, together with the fact that $\tilde{\delta }\le \bar{\phi }^n \le 1$ uniformly in *n*, and applying the Aubin-Lions compactness lemma Lions (1969), we obtain the strong convergence of $\{c_s^n\}$ in $L^2((0,T)\times \Omega )$.

Considering equations for $\phi ^n(t, x+ h)-\phi ^n(t,x)$ and $c_b^n(t, x+ h) - c_b^n(t,x)$ and taking $\phi ^n(t,x+h) - \phi ^n(t,x)$ and $c_b^n(t,x+h)(1- \phi ^n(t, x+ h)) - c_b^n(t,x) (1- \phi ^n(t,x))$ as test functions respectively, we obtain

$$\begin{aligned}&\big \Vert c_b^n(t,\cdot +h)(1-\phi ^n(t, \cdot +h)) - c_b^n(t)(1- \phi ^n(t))\big \Vert ^2_{L^2(\Omega _h)} \\ &+ \big \Vert \phi ^n(t,\cdot +h) - \phi ^n(t)\big \Vert _{L^2(\Omega _h)}^2 \le \big \Vert \phi _0(\cdot +h) - \phi _0\big \Vert ^2_{L^2(\Omega _h)}\\ &\qquad + \big \Vert c_{b,0}(\cdot +h)(1-\phi _0(\cdot +h)) - c_{b,0}(1- \phi _0) \big \Vert ^2_{L^2(\Omega _h)} \\ &+ C \int _0^t\Big [\big \Vert \phi ^n(\tau )(c_s^n(\tau ,\cdot +h) - c_s^n(\tau ))\big \Vert _{L^2(\Omega _h)}^2 + \big \Vert \phi ^n(\tau ,\cdot +h) - \phi ^n(\tau )\big \Vert _{L^2(\Omega _h)}^2 \quad \\ &\qquad + \big \Vert c_b^n(\tau ,\cdot +h)(1-\phi ^n(\tau , \cdot +h)) - c_b^n(\tau )(1- \phi ^n(\tau ))\big \Vert ^2_{L^2(\Omega _h)} \Big ] d\tau , \end{aligned}$$

for some $h \in \mathbb R^d$, where $\Omega _h = \{ x \in \Omega : \textrm{dist}(x, \partial \Omega ) >|h| \}$. Then, using the estimate

$$ \int _0^t\Vert \phi ^n(\tau )(c_s^n(\tau ,\cdot +h) - c_s^n(\tau ))\Vert _{L^2(\Omega _h)}^2 d\tau \le C_1 h^2 \Vert \phi ^n \nabla c_s^n\Vert ^2_{L^2((0,T)\times \Omega )} \le C h^2, $$

where the constant $C>0$ depends on *T*, $\tilde{\delta }$, $M_b$, and $M_s$, and is independent of *n*, due to uniform in *n* boundedness of $\phi ^n$ and $\bar{\phi }^n\ge \tilde{\delta }$, and applying the Gronwall lemma yields

25 $$\begin{aligned} \begin{aligned}\sup \limits _{t \in (0,T)}\Vert c_b^n(t,\cdot +h)(1-\phi ^n(t, \cdot +h)) - c_b^n(t)(1- \phi ^n(t))\Vert ^2_{L^2(\Omega _h)} \\\quad +\sup \limits _{t \in (0,T)} \Vert \phi ^n(t,\cdot +h) - \phi ^n(t)\Vert _{L^2(\Omega _h)}^2\le \sigma (h), \end{aligned} \end{aligned}$$

where $\sigma (h) \rightarrow 0$ as $h\rightarrow 0$, uniformly in *n*. This, together with estimate (21) for the time derivatives and boundedness of $\phi ^n$ and $c_b^n(1-\phi ^n)$, ensures the equicontinuity and hence the strong convergence of the sequences $\{\phi ^n\}$ and $\{ c_b^n(1-\phi ^n)\}$ in $L^2((0,T)\times \Omega )$. The property $\tilde{\delta }\le \phi (t,x) \le 1$ for $(t,x) \in (0,T)\times \Omega$ follows directly from the equation for $\phi$ in (17) and the assumptions on the initial conditions.

Thus we have that *K* maps *V* into itself and compactness is ensured by the strong convergence results. Applying the Schauder fixed point theorem we obtain existence of solution of model (9), (11).

To show uniqueness, we consider the equations satisfied by the difference of two solutions and using energy arguments, similar as in the derivation of estimates (20), obtain

26 $$\begin{aligned} \begin{aligned} \Vert \phi _1(t)- \phi _2(t)\Vert ^p_{L^p(\Omega )} + \Vert c_{b,1}(t)(1-\phi _1(t))- c_{b,2}(1-\phi _2(t))\Vert ^p_{L^p(\Omega )}\quad \\ \le C_p \int _0^t \Vert c_{s,1} (\tau )- c_{s,2}(\tau )\Vert ^p_{L^p(\Omega )} d\tau , \end{aligned} \end{aligned}$$

for $p\ge 2$. Considering $\partial _t c_s$ as test function in equations for $c_s$ in (14), together with a regularisation by a mollifier, see e.g. Showalter (1996), and assumptions on $c_{s, 0}$ and $D_s(\phi )$, yields

$$\begin{aligned}&\Vert \partial _t c_s \Vert ^2_{L^2((0,T)\times \Omega )} + \sup \limits _{(0,T)} \Vert \sqrt{d(\phi ) }\nabla c_s \Vert ^2_{L^2(\Omega )} \\&\quad \le C_1 +C_2 \Vert \partial _t \phi \Vert _{L^\infty ((0,T)\times \Omega )} \Vert \nabla c_s\Vert ^2_{L^2((0,T)\times \Omega )} \le C. \end{aligned}$$

This, together with the $L^p$-regularity for elliptic problems in $C^{1,1}$ or convex domains, yields

$$ \Vert c_s\Vert _{L^2(0,T;W^{1,p}(\Omega ))} \le C, \quad 3<p\le 7, $$

see e.g. Dauge (1992). For two solutions $c_{s,1}$ and $c_{s,2}$ we have

27 $$\begin{aligned} \begin{aligned}&\partial _t \Vert c_{s,1}(t) - c_{s,2}(t)\Vert _{L^2(\Omega )}^2 + \Vert \sqrt{d(\phi _1(t))} \nabla (c_{s,1} (t)- c_{s,2}(t))\Vert _{L^2(\Omega )}^2\\ &\quad \le C \Big [ \Vert c_{s,1}(t) - c_{s,2}(t)\Vert _{L^2(\Omega )}^2 +\Vert \nabla c_{s,2}(t)\Vert ^2_{L^4(\Omega )} \Vert \phi _1(t) - \phi _2(t)\Vert _{L^4(\Omega )}^2 \\ &\qquad \qquad + \Vert \phi _1(t) - \phi _2(t)\Vert _{L^2(\Omega )}^2 \Big ]. \end{aligned} \end{aligned}$$

Using (26) and the estimate

$$ \Vert v\Vert _{L^4(\Omega )} \le C \left[ \Vert \nabla v \Vert ^\theta _{L^2(\Omega )} \Vert v \Vert ^{1-\theta }_{L^2(\Omega )} + \Vert v\Vert _{L^2(\Omega )}\right] , $$

with $\theta = d/4$ and $d=2,3$, which follows from the Gagliardo-Nirenberg interpolation inequality, and integrating (27) with respect to the time variable, we obtain

$$\begin{aligned}&\Vert c_{s,1} (t)- c_{s,2}(t)\Vert _{L^2(\Omega )}^2 + 2\Vert \sqrt{d(\phi _1)} \nabla (c_{s,1} - c_{s,2})\Vert _{L^2((0,t)\times \Omega )}^2\\&\quad \le \varsigma \Vert \nabla c_{s,1} - \nabla c_{s,2}\Vert _{L^2((0,t)\times \Omega )}^2 + C \Vert c_{s,1} - c_{s,2}\Vert _{L^2((0,t)\times \Omega )}^2, \end{aligned}$$

for $t \in (0,T]$. Choosing a sufficiently small $0<\varsigma \le 2 \tilde{\delta }$ and applying the Gronwall inequality yields $c_{s,1} = c_{s,2}$ a.e. in $(0,T)\times \Omega$. Then estimate (26) implies $\phi _1 = \phi _2$ and $c_{b,1}=c_{b,2}$ a.e. in $(0,T)\times \Omega$, and hence uniqueness of solutions of model (9), (11).

The regularity $\phi \in L^\infty (0,T; C(\overline{\Omega }))$ required for the results in Dauge (1992) is ensured by combining a regularisation argument, the fact that $W^{1,p}(\Omega ) \subset C^{0, \alpha }(\overline{\Omega })$ for $p>d$ and $\alpha = 1 - d/p$, and the estimate

$$\begin{aligned} \Vert \nabla \phi (t)\Vert _{L^p(\Omega )}&\le C \Big (\Vert \nabla \phi _0 \Vert _{L^p(\Omega )} + \Vert \nabla (c_{b,0} (1-\phi _0) )\Vert _{L^p(\Omega )} \\ &\quad + \Big \Vert \int _0^t |\nabla c_s(\tau )| d\tau \Big \Vert _{L^p(\Omega )} \Big ) \; \text{ for } t \in (0,T]. \end{aligned}$$

The last estimate follows from applying the Gronwall inequality and taking the $L^p(\Omega )$-norm in the inequality

$$\begin{aligned} |\nabla \phi (t,x)| &  \le |\nabla \phi _0(x)| + C_1 |\nabla (c_{b,0}(x)(1- \phi _0(x)))|\\ &  + C_2\int _0^t\big (|\nabla c_s(\tau ,x)| + |\nabla \phi (\tau ,x) | \big ) d \tau , \end{aligned}$$

for $(t,x) \in (0,T]\times \Omega$, which in turn is obtained from the equations for $\phi$ and $c_b(1- \phi )$ in (9).$\square$

### Remark 4.2

Using similar arguments as in the proof of Theorem 4.3 we can also show the existence and uniqueness of solutions $\phi \in H^1(0,T; L^2(\Omega ))$, $c_s \in L^\infty (0,T; L^2(\Omega )) \cap L^2(0,T; H^1(\Omega ))$, $c_b(1-\phi ) \in L^\infty (0,T; L^2(\Omega \times \Gamma )) \cap L^2((0,T)\times \Omega ; H^1(\Gamma ))$, with $\partial _t c_s \in L^2(0,T; H^1(\Omega )^\prime )$, $\partial _t(c_b(1-\phi )) \in L^2((0,T)\times \Omega ; H^1(\Gamma )^\prime )$, to the two-scale model (13), satisfying

$$ 0\le \phi (t,x) \le 1, \quad 0 \le c_b(t,x,y)(1- \phi (t,x)) \le M_b, \quad 0 \le c_s(t,x) \le M_s , $$

for $$x \in \Omega $$, $$y \in \Gamma $$, and $$t\ge 0$$, where $$M_b= \max \{M_{b,0}, {\kappa _b}/{\beta _b}\}$$ and $$M_s= \max \{M_{s,0}, {\kappa _s}/{\beta _s}\}$$, and the lower bound (16) for $$t \in [0,T]$$. Notice that $$\phi $$ is independent of $$y \in \Gamma $$ and we can use $$(1-\phi )c_b$$ as a test function in the equation for $$c_b$$ in (13). Additionally we have that  satisfies the same ODE equation as $$ c_b (1- \phi )$$ in (9). More specifically, integrating equation for $$c_b $$ in (13) over $$\Gamma $$ yieldswhere we used that the integral over $$\Gamma $$ of $$\nabla _{\Gamma , y} \cdot (D_b(1-\phi ) \nabla _{\Gamma , y} c_b)$$ is zero.

## Finite element approximation

In this section we describe a numerical method for the approximation of the macroscopic model formulated in section 3.1. We do not present results for the approximation of the two-scale model as under the simplified intracellular processes considered in this study cell-scale concentrations are effectively spatially uniform at the timescales of invasion. A number of recent cell-scale models for cellular signalling processes demonstrate inhomogeneous membrane concentrations typically by modelling detailed biochemistry on the membrane or within the cell e.g., Levine and Rappel (2005); Neilson et al. (2011); Jilkine and Edelstein-Keshet (2011); Garcke et al. (2016). Our framework readily allows for such complex intracellular dynamics to be incorporated and simulated which we intend to consider in future work. In particular, developing a model for the intracellular signalling processes which lead to the production of MMPs, both soluble and membrane bound, by cancer cells is an interesting direction for future studies. The numerical approximation of the two-scale model is described in detail in Ptashnyk and Venkataraman (2020); Lakkis et al. (2024).

We define computational domains ${\Omega }_h$ and $Y_{h,e}$ by requiring that ${\Omega }_h$ and $Y_{h,e}$ are polyhedral approximations to ${\Omega }$ and $Y_e$ respectively. We assume that ${\Omega }_h$ and $Y_{h,e}$ consist of the union of *d* dimensional simplices (triangles for $d=2$ and tetrahedra for $d=3$).

We define ${\mathcal {S}_{h,{\Omega }}}$ and ${\mathcal {S}_{h,e}}$ to be triangulations of ${\Omega }_h$ and $Y_{h,e}$ respectively and assume that each consists of closed non-degenerate simplices and that the triangulations are conforming. We define piecewise linear finite element spaces as follows

$$\begin{aligned} {\mathbb {V}_{h,\Omega }}&=\left\{ \Phi \in C({\Omega }_h) : \; \Phi \vert _k\in \mathbb {P}^1,\quad \forall k\in {\mathcal {S}_{h,{\Omega }}}\right\} ,\\ {\mathbb {V}_{h,e}^\#}&=\left\{ \!\Phi \in \operatorname {H}^{1}_\mathrm{{ per}}(Y_{h,e})\cap C(Y_{h,e}){\text { with }\int _{Y_{h,e}}\Phi \, dx=0} : \;\Phi \vert _k\in \mathbb {P}^1,\quad \forall k\in {\mathcal {S}_{h,e}}\!\right\} , \end{aligned}$$

where $\operatorname {H}^{1}_\mathrm{{ per}}(Y_{h,e})$ denotes the subspace of *Y*-periodic functions in $\operatorname {H}^{1}(Y_{h,e})$. The numerical scheme for the solution of the cell problems to compute the diffusion tensor $D_s^\textrm{hom}$ is, for $j=1,\dots ,d$, determine $W^j\in {\mathbb {V}_{h,e}^\#}$ such that

28 $$\begin{aligned}&\left\langle \bar{D}_s(\nabla _yW^j+ e_j),\nabla _y \Phi \right\rangle _{Y_{h,e}} =0, \end{aligned}$$

for all $$\Phi \in {\mathbb {V}_{h,e}^\#}$$.

For the approximation of solutions of the macroscopic model (14), (11) we employ an IMEX scheme (Ruuth 1996). We consider a uniform (purely for simplicity) partition of the time interval [0, *T*] into *N* subintervals with step size $\tau =T/N$. The scheme is defined as follows:

For $n=1,\dots ,N$ and given $(\Phi ^{n-1}, C_s^{n-1}, C_b^{n-1}, S^{n-1})\in ({\mathbb {V}_{h,\Omega }})^4$ find $(\Phi ^{n}, C_s^{n}, C_b^{n}, S^{n})\in ({\mathbb {V}_{h,\Omega }})^4$ such that

29 $$\begin{aligned} \begin{aligned}&\left\langle \Phi ^n,\Psi _1\right\rangle _{\Omega _{h}}=\left\langle \Phi ^{n-1},\Psi _1\right\rangle _{\Omega _{h}}+\tau \left\langle -(1-S^{n-1})\mu _s\Phi ^{n-1}C_s^{n-1},\Psi _1\right\rangle _{\Omega _{h}}\\&\qquad +\tau \left\langle {-}(1-S^{n-1})\mu _b\Phi ^{n-1}C_b^{n-1}(1-\Phi ^{n-1}),\Psi _1\right\rangle _{\Omega _{h}},\\&\left\langle C_s^n,\Psi _2\right\rangle _{\Omega _{h}}+\tau \left\langle D_s({\Phi ^{n})\nabla C_s^{n}},\nabla \Psi _2\right\rangle _{\Omega _{h}}\\&\quad =\left\langle C_s^{n-1},\Psi _2\right\rangle _{\Omega _{h}}+\tau \left\langle \kappa _sf_s(C_s^{n-1})(1-\Phi ^{n-1}),\Psi _2\right\rangle _{\Omega _{h}}\\&\qquad +\tau \left\langle -((1-S^{n-1})\mu _s\Phi ^{n-1}+\beta _s)C_s^{n-1},\Psi _2\right\rangle _{\Omega _{h}},\\&\left\langle C_b^n(1-{\Phi ^{n}}),\Psi _3\right\rangle _{\Omega _{h}}\\&\quad =\left\langle {C_b^{n-1}}(1-\Phi ^{n-1}),\Psi _3\right\rangle _{\Omega _{h}}+\tau \left\langle \kappa _bf_b(\Phi ^{n-1})(1-\Phi ^{n-1}),\Psi _3\right\rangle _{\Omega _{h}}\\&\qquad +\tau \left\langle -\left( \left( 1-S^{n-1}\right) \mu _b\Phi ^{n-1}+\beta _b\right) C_b^{n-1}(1-\Phi ^{n-1}),\Psi _3\right\rangle _{\Omega _{h}}, \\&\left\langle S^n,\Psi _4\right\rangle _{\Omega _{h}} =\left\langle S^{n-1},\Psi _4\right\rangle _{\Omega _{h}}+\tau \left\langle -\delta _s(1-\Phi ^{n-1})C_b^{n-1}S^{n-1},\Psi _4\right\rangle _{\Omega _{h}}, \end{aligned} \end{aligned}$$

for all $\Psi _i\in {\mathbb {V}_{h,\Omega }}, i=1,2,3,4$. The initial approximations at $t=0$ are taken to be the linear Lagrange interpolants of the initial data. The scheme for model (9), (11) without matrix suitability corresponds to (29) with $S^n=0$ for all *n*, hence only three equations are solved per time step. If greater accuracy is desired in terms of linearisation a fixed-point iteration or even a Newton-type linearisation could be employed as has been considered for semilinear reaction-diffusion systems in previous works, eg. Madzvamuse and Chung (2014). Our previous studies of such systems suggest the IMEX method we employ above is robust as the diffusive terms, even when treated implicitly as above, necessitate the use of a small time-step (Lakkis et al. 2013).

## Model parametrisation and simulation results

### Parametrisation of the model equations and choice of discretisation parameters

To facilitate comparison with the results in Deakin and Chaplain (2013), for the parameters in the macroscopic models (9), (11) and (14), (11) in section 3 we use the parameter values of Deakin and Chaplain (2013), see Table 1. It is a worthwhile direction for future work to investigate the sensitivity of the numerical solutions to changes in the parameter values and to understand whether the broad conclusions are robust to variations in the parameters. We checked that the simulation results reported on below remain qualitatively indistinguishable from those obtained using numerical parameters (mesh-size and where relevant time step) approximately double those used to generate the simulations reported on in the manuscript.

**Table 1:** Parameter values used for numerical simulations for models (9), (11) and (14), (11)

	non-dimensional values	original values	references
$\Omega$	$(0,2)^d$, $d=2,3$	$0.1 \textrm{cm}$	Collier et al. (2011)
$\kappa _s$	4	$4\cdot 10^{-4} \textrm{s}^{-1}$	estimated, Deakin and Chaplain (2013)
$\kappa _b$	5	$5\cdot 10^{-4} \textrm{s}^{-1}$	estimated, Deakin and Chaplain (2013)
$D_s$	$1.29\cdot 10^{-2}$	$1.29 \cdot 10^{-8} \, \textrm{cm}^2 \textrm{s}^{-1}$	Collier et al. (2011)
$\mu _b$, $\mu _s$	1	−	Anderson et al. (2000)
$\mu _b$, $\mu _s$	8.15	−	Chaplain and Lolas (2005)
$\beta _s$, $\beta _b$	0.1	$1\cdot 10^{-5} \textrm{s}^{-1}$	estimated, Anderson et al. (2000); Deakin and Chaplain (2013)
$\delta _s$	1	$-$	Deakin and Chaplain (2013)

### Approximation of the effective diffusion tensor for soluble MMPs

As discussed in section 3 we obtain an effective diffusion tensor for different volume fractions of the ECM by solving problems (8) on different geometries corresponding to different volume fractions of ECM and fitting, using least squares, a polynomial based on these values.

In the $2\textrm{d}$ case the domains we consider correspond to squares of the form $[0,L]^2$ with $n^2$ circle(s) of radius $r=\frac{3}{40}L$, with $n=1,2,3,4,5,6$, removed, i.e., the cells are circular. The centres of the circles are taken to form a square lattice, with the resulting geometry as shown in Figure 2. The corresponding volume fractions of ECM are 0.982, 0.929, 0.841, 0.717, 0.558 and 0.364 for $n=1,2,3,4,5$ and 6 respectively.

For the $3\textrm{d}$ case we adopt a similar geometric setup to the $2\textrm{d}$ case using cubes of the form $[0,L]^3$ with $n^3$ spheres of radius $r=\frac{3}{25}L$, with $n=1,2,3,4$, removed, i.e., the cells are spherical. The centres of the spheres are taken to form a cubic lattice with the resulting geometry shown in Figure 3. The corresponding volume fractions of ECM are 0.993, 0.942, 0.805 and 0.537 for $n=1,2,3$ and 4 respectively.

For the choices of microscopic geometry described above, we note that, due to symmetry, the effective diffusion tensor will be isotropic and diagonal. This is reflected in the numerical results, for which an unstructured triangulation is used, in that the off diagonal components of the computed diffusion tensors for all geometries are two orders of magnitude smaller than the diagonal components and the diagonal components of the computed diffusion tensors on each specific geometry exhibit only small differences ($<1\%$). We therefore obtain values for fitting an effective diffusion coefficient by assuming we have a $\phi$ dependent scaling of the diffusion tensor and take the average of the diagonal components of the computed diffusion tensors to approximate this scaling on each microscopic geometry. We take $D_s(0)=0$ as a further fitting point as we assume the soluble MMPs do not diffuse through the cancer cells. We use a finite element method implemented in the FEniCS software (Logg et al. 2012) for the approximation of (28). In the simplest setting, where the effective diffusion tensor is diagonal and isotropic, we compute a least squares polynomial fit to obtain the effective diffusivity in $2\textrm{d}$ and $3\textrm{d}$ (in the examples below a cubic polynomial appears adequate to fit the computed data). The fitted polynomials obtained are given by

30 $$\begin{aligned} {D_s(\phi )}=\big (0.25\phi ^3+0.33\phi ^2+0.42\phi \big ){D_s(1)}, \end{aligned}$$

in $$2\textrm{d}$$ and

31 $$\begin{aligned} {D_s(\phi )}=\big (0.89\phi ^3-2.35\phi ^2+2.46\phi \big ){D_s(1)}, \end{aligned}$$

in $3\textrm{d}$. Figure 4 shows plots of the effective diffusivity as the volume fraction of ECM changes.

We note that the effective diffusivity appears to be a convex function of the volume fraction in $2\textrm{d}$ but not in $3\textrm{d}$. We note that heuristic formulae for the effective diffusivity of a porous medium as a function of volume fraction of the medium versus void are given in many previous works e.g., Weissberg (1963); Du Plessis and Woudberg (2009) where various (convex) functions are proposed. We note that more complex dependence on volume fraction including concave functions are computed in Tallarek et al. (2019)(c.f., Figures 11 and 14) albeit in a significantly more complicated setup. The result above may also be linked to interesting results in percolation theory such that the critical exponents that relate diffusivity of the medium with volume fraction are dimension dependent (Bunde and Kantelhardt 2005). This is an interesting direction for future work. To investigate further how the effective diffusivity varies depending on the choice of microstructure, in Appendix A.1, we consider a different geometric setup for the microstructure and the effective diffusivities computed are quantitatively comparable with those presented above. For the general case of a non-diagonal effective diffusion tensor, simple polynomial fits or affine interpolation may not be sufficient as positive definiteness can in general not be ensured. This is discussed in Appendix 7.1 wherein geometries that yield a full effective diffusion tensor are considered. An interesting area for future work would be to consider if those results are robust to irregular shape and distribution of cells within the microstructure.

### Simulation of the macroscopic invasion model (9), (11)

We report on simulations of the macroscopic invasion model (9), (11) using the parameters in Table 1 in section 6.1. To understand the relative contributions of membrane bound and soluble MMPs we run three simulation scenarios in $2\textrm{d}$ and $3\textrm{d}$, i.e., six in total. One where both soluble and membrane bound MMPs degrade the ECM, one where only soluble MMPs degrade the ECM ($\mu _b=0$) and one where only membrane bound MMPs degrade the ECM ($\mu _s=0$).

For the simulations in $2\textrm{d}$ we take $\Omega =[-1,1]^2$ and the initial condition

32 $$\begin{aligned} \phi _0(x)=\Big (1-e^{-\left( (4x_1)^2+(8x_2)^2\right) }\Big ), \end{aligned}$$

and the initial data for the remaining variables are set to 0. We take the timestep $\tau =10^{-2}$ and use a mesh with 12909 degrees of freedom (DOFs).

**Fig. 5:**
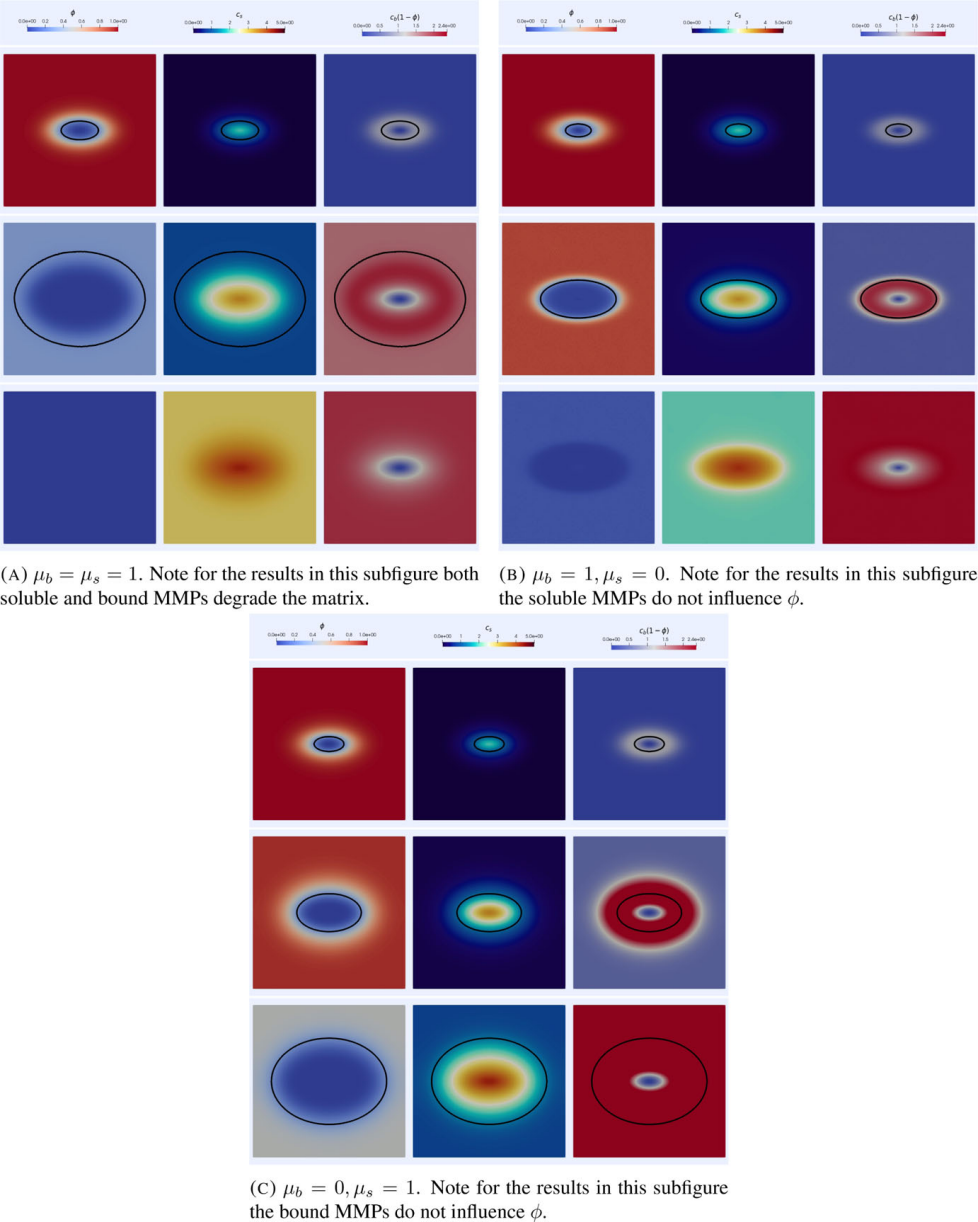
Simulations of the invasion model (9), (11) in $2\textrm{d}$. In each subfigure, each row corresponds to times $t=1, 3$ and 5. The black contour indicates the level set $\phi =0.25$ corresponding to a volume fraction of $75\%$ cancer cells. The effect of membrane bound MMPs increasing the speed of invasion is apparent whilst the degradation of the ECM by the soluble MMPs appears to generate more radially symmetric invasive profiles. Parameter values as in Table 1 (Color figure online)

Figure 5 shows the results of simulations in $2\textrm{d}$. We observe MMP mediated invasion by cancer cells. In the case where both membrane bound and soluble MMPs degrade the ECM, see Figure 5a, we observe rapid invasion of the ECM and, at least at the initial stages of invasion, the membrane bound MMPs remain localised to a region close to level set $\phi =0.25$. The soluble MMPs are primarily located in the region where the volume fraction of cancer cells is high and the influence of slowed transport into the ECM due to the $\phi$ dependence of the diffusivity is evident. In the simulations where the soluble MMPs are unable to degrade the ECM, see Figure 5b, we still observe relatively rapid invasion, albeit with reduced speed compared to the case where both MMP species degrade the ECM. The profile of the invasive front appears to match the initial shape of the invasive front. In the simulations where the bound MMPs are unable to degrade the ECM, see Figure 5c, we observe the slowest invasive speed of all the simulations reported in Figure 5. At the final time $t=5$ this is the only case in which the entirety of the domain does not consist of at least $75\%$ cancer cells. The invasive front appears to take on a more radially symmetric shape similar to the case when both species of MMP degrade the matrix. As a whole the results depicted in Figure 5 illustrate that the model reflects behaviour observed in experiments such as (Sabeh et al. (2009), Figure 1A), i.e., the bound MMPs are more significant for cancer invasion into the ECM via matrix degradation than the soluble MMPs.

For the simulations in $3\textrm{d}$ we take $\Omega =[-1,1]^3$ and the initial condition

33 $$\begin{aligned} \phi _0(x)=\Big (1-e^{-\left( (4x_1)^2+(4x_2)^2+(8x_3)^2\right) }\Big ), \end{aligned}$$

and the initial data for the remaining variables are set to 0. We take the time step $\tau =10^{-2}$ and use a mesh with 61905 DOFs.

**Fig. 6:**
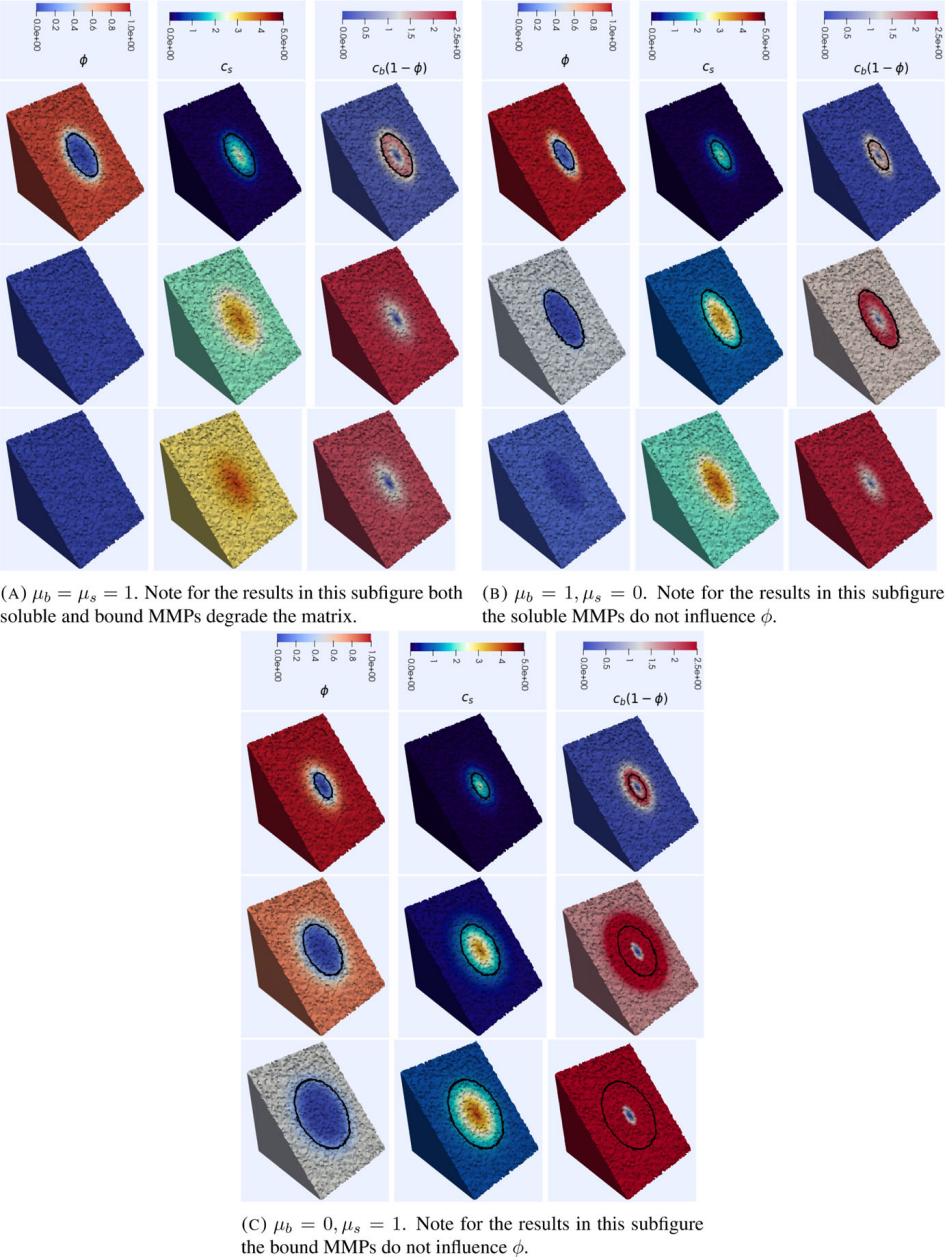
Simulations of the invasion model (9), (11) in $3\textrm{d}$. In all plots we have half of the domain transparent to aid visualisation. In each subfigure, each row corresponds to times $t=2, 4$ and 5. The black contour indicates the level set $\phi =0.25$ corresponding to a volume fraction of $75\%$ cancer cells. The results appear analogous to the $2\textrm{d}$ case despite the relatively higher diffusivity of soluble MMPs for $\phi >0$. The effect of membrane bound MMPs increasing the speed of invasion is apparent whilst the degradation of the ECM by the soluble MMPs appears to generate more radially symmetric invasive profiles. Parameter values as in Table 1 (Color figure online)

Figure 6 shows results of $3\textrm{d}$ simulations. The results are analogous to the $2\textrm{d}$ simulations, see Figure 5, and hence we infer that in this parameter regime the model exhibits similar behaviour in $2\textrm{d}$ and $3\textrm{d}$ despite the qualitatively different effective diffusivities. In the following sections of the manuscript, we therefore report only on $2\textrm{d}$ simulations and expect that analogous results would be obtained for the corresponding $3\textrm{d}$ simulations.

### Simulations of the model with varying matrix suitability (14), (11)

We now report on simulations of the model (14), (11) with varying matrix suitability for invasion. For all simulations in this section we take $\Omega =[-1,1]^2$, the time step $\tau =10^{-2}$ and use a mesh with 12909 DOFs. We first consider initial conditions as in the $2\textrm{d}$ simulations of section 6.3 along with initial conditions for *s* of the form

34 $$\begin{aligned} s_0(x)=1-0.1\left( \cos (4\pi x_1)\cos (4\pi x_2)\right) ^2, \end{aligned}$$

which corresponds to a matrix that is largely unsuitable for invasion at the initial time.

**Fig. 7:**
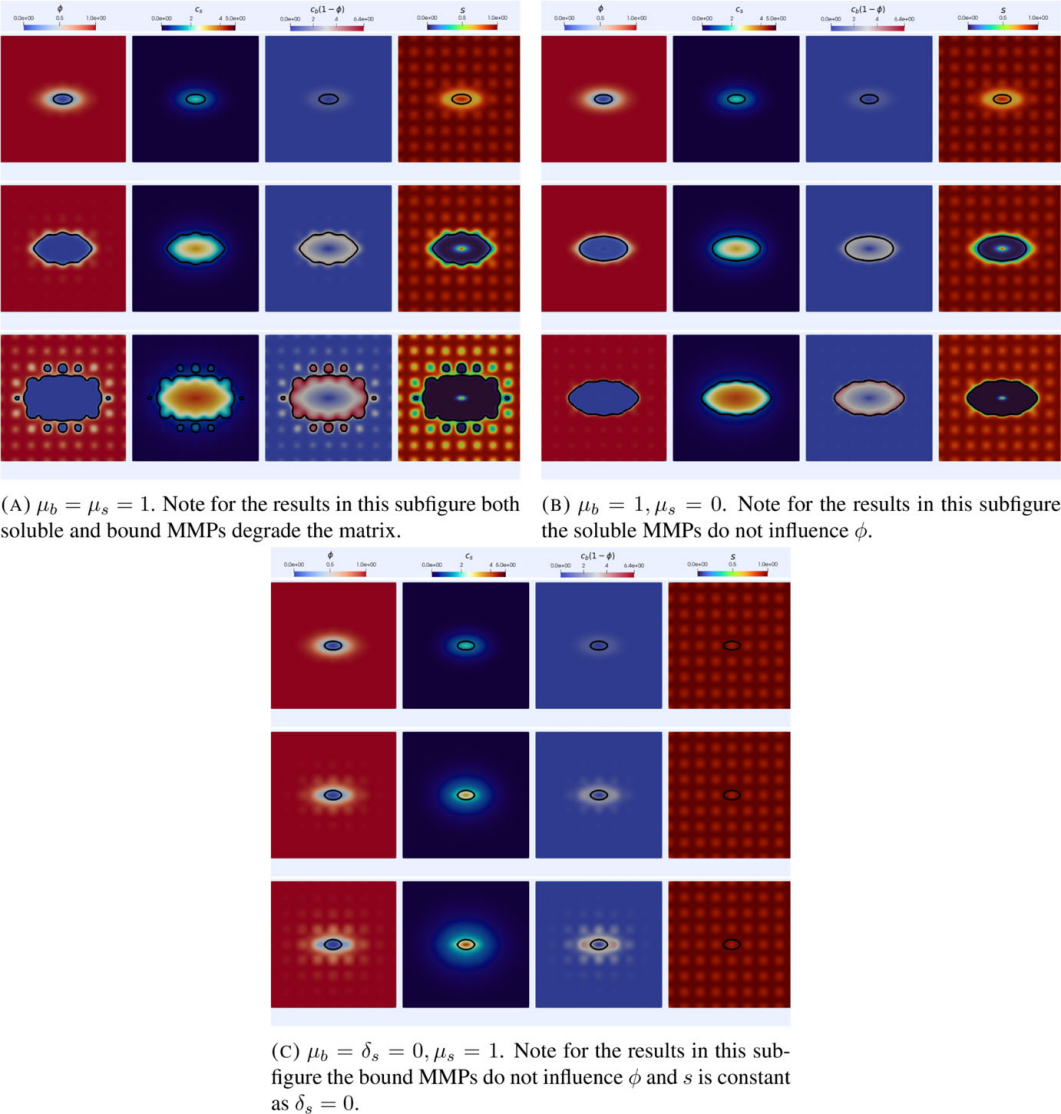
Simulations of the heterogeneous ECM suitability invasion model (14), (11) in $2\textrm{d}$ with initial conditions corresponding to low matrix suitability (34). In each subfigure, each row corresponds to times $t=1, 3$ and 5. The black contour indicates the level set $\phi =0.25$ corresponding to a volume fraction of $75\%$ cancer cells. We note that, in both cases where the bound enzymes affect suitability, spatial inhomogeneities arise in the invasive front. We also observe that the invasiveness is greatly reduced in the case where the bound MMPs do not degrade the ECM or influence its suitability. Parameter values as in Table 1 (Color figure online)

Figure 7 shows the corresponding simulation results. We note that in both cases where the bound MMPs affect the suitability (Figures 7a and 7b) the ECM is made more suitable for invasion and then the ECM is degraded with the matrix suitability close to 0 in the regions where $\phi <0.25$. On the other hand in the simulation results in Figure 7c where the bound MMPs do not degrade the ECM or change the suitability (hence *s* is constant in time) the invasive process is almost completely stalled with virtually no change in the level set corresponding to $\phi =0.25$. As noted previously when discussing the simulations with a homogeneous ECM these results are consistent with the experimental observations of [Sabeh et al. (2009), Figure 1A].

For the final set of simulation results we consider a setup that is similar to that of (Deakin and Chaplain (2013), Figure 7) and take initial conditions of the form

35 $$\begin{aligned} \begin{aligned} \phi _0(x)=1-e^{-4(1+x_1)}\quad \text {and}\quad s_0(x)=\frac{1}{2}\left( 1+\cos (4\pi x_1)\cos (4\pi x_2)\right) , \end{aligned} \end{aligned}$$

and set the initial conditions for the MMPs to 0. We note the initial conditions correspond to cancer cells invading from a region near the left boundary and a checkerboard initial suitability with suitable and unsuitable regions for invasion both present at the initial time.

**Fig. 8:**
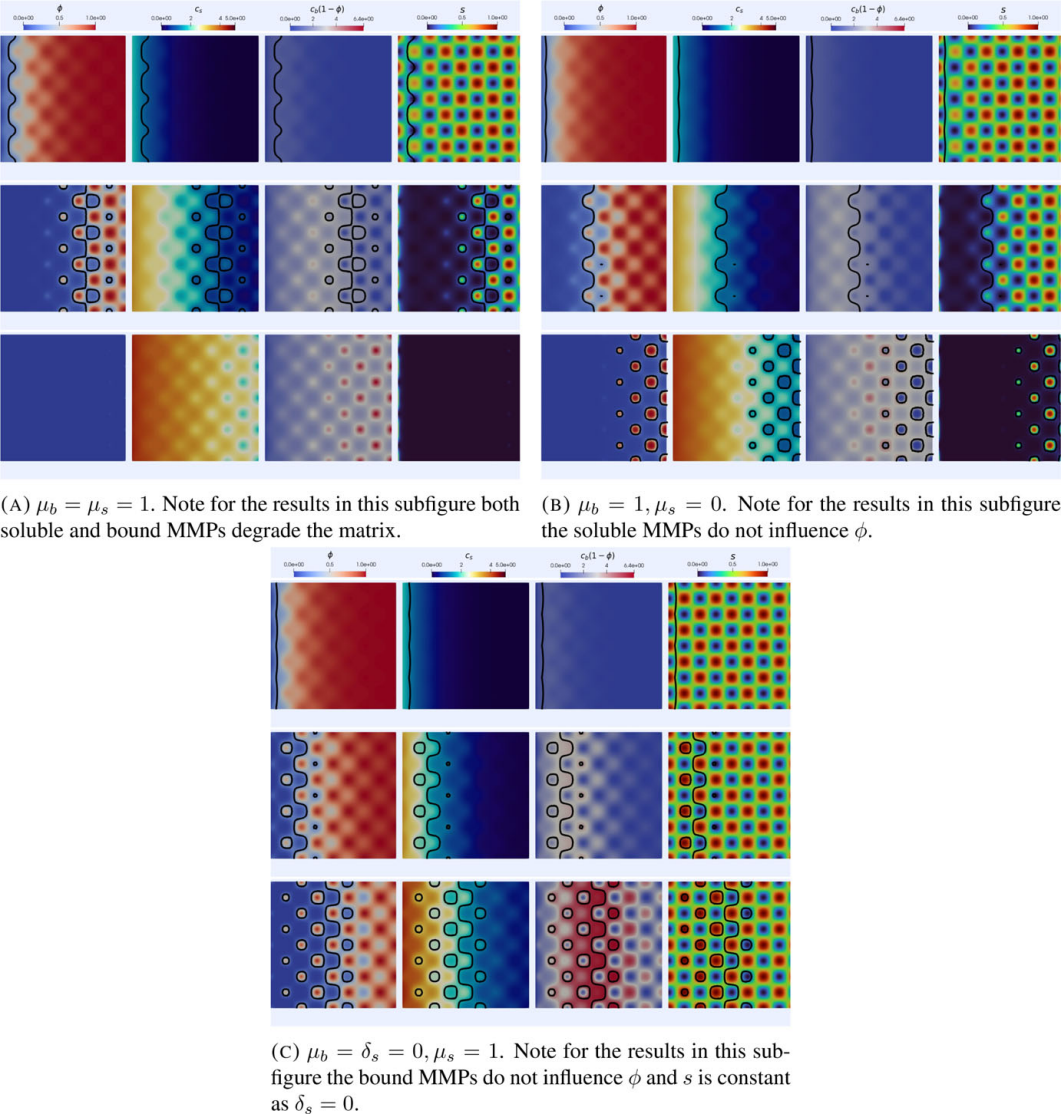
Simulations of the heterogeneous ECM suitability invasion model (14), (11) in $2\textrm{d}$ to be compared with (Deakin and Chaplain (2013), Figure 7). In each subfigure, each row corresponds to times $t= 1, 3$ and 5. The black contour indicates the level set $\phi =0.25$ corresponding to a volume fraction of $75\%$ cancer cells. The role of the bound MMPs in making the matrix more suitable for invasion is clear as is the reduced invasiveness in the case where the bound MMPs do not degrade the ECM or influence its suitability. Parameter values as in Table 1. (Color figure online)

The results of the simulation are reported on in Figure 8 and are broadly similar to those shown in (Deakin and Chaplain (2013), Figure 7), and exhibit similar qualitative features to those reported on in Figure 7. We observe a heterogeneous invasion front in all cases that largely matches the spatial variations in the suitability at the initial time. In the simulations where the bound MMPs are active (Figures 8a and 8b) we again observe that the suitability is close to zero in regions where $\phi <0.25$. We also note that despite the presence of regions which are suitable for invasion at the initial time, the invasive speed, in the case where the bound MMPs are not active, is reduced (Figure 8c), albeit not as much as was the case in the simulations of Figure 7.

## Discussion and possible extensions

In this work, we have presented a multiscale framework for the modelling of cancer cell invasion into the ECM through the degradation of the ECM by bound and soluble matrix-degrading enzymes. Our framework is novel in that we give a rational way of encoding microscopic details in a macroscopic (tissue-level) model, which accounts for the computation of an effective diffusivity that depends on the volume fraction of the ECM as well as a means of incorporating details of cell-scale signalling processes in a tissue-level framework. We report on numerical simulations for the macroscopic model, which illustrate the role bound and soluble enzymes play in the invasion process and highlight interesting features such as the propensity of degradation of the ECM by membrane-bound enzymes to generate more spatially heterogeneous invasion profiles. The latter could be of importance in understanding the onset of complex tumour morphologies, which are associated with metastasis.

One main area for future work is the rigorous derivation of a macroscopic invasion model from microscopic models such as those proposed in this work, which is of significant interest beyond this specific application. We believe this should be possible in the setting where cell movement drives invasion but proliferation is neglected, using the ideas from the multiscale analysis of free boundary problems, e.g. Kim and Mellet (2010); Pozár (2015); Rodrigues (1982); Visintin (2007). As mentioned briefly previously, incorporating proliferation even at the microscopic level appears to require the development of a novel theory of continuum PDE models beyond topological changes, and this needs to be clarified before the derivation of macroscopic models involving proliferation can be considered.

Several other extensions of this study are important to develop a detailed description and a better understanding of cancer invasion. From the modelling perspective, the two-scale framework warrants further study, especially if a detailed cell-signalling model such as those proposed in Ptashnyk and Venkataraman (2020) with non-trivial dynamics is considered. The structure of the ECM can be represented in greater detail, such as accounting for the orientation of the collagen fibres, which influence cell migration as well as a number of other factors, see Crossley et al. (2024) for a review. Depending on the timescales, ECM remodelling could also be included. The mixture-model-based formalism we employ could also be extended to allow for necrotic regions within the tumour. From the perspective of analysis, an obvious extension would be to extend the proof of the well-posedness to the degenerate case where we do not assume a strictly positive lower bound for the initial condition for the volume fraction of ECM. However, this appears challenging and requires the development of novel analytical techniques. From the applications perspective, the results above complement the work of Deakin and Chaplain (2013) by allowing for greater connection to microscopic cell scale models of matrix degradation. The work of Deakin and Chaplain (2013) was seeking to model the experiments of Sabeh et al. (2009). Further numerical experiments in which we simulate more closely the scenarios considered in the experiments of Sabeh et al. (2009) and other related studies will allow us to assess the ability of the mathematical model to reproduce biological observations with the eventual goal of augmenting and guiding biological experiments through computational simulations.

## Figures and Tables

**Fig. 9:**
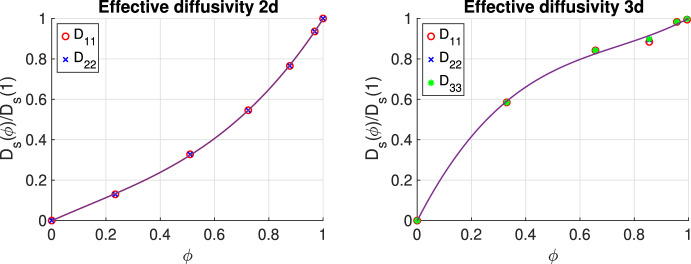
Fitted effective diffusivity in $2\textrm{d}$ and $3\textrm{d}$ for a microstructure consisting of a single square (cube in $3\textrm{d}$) shaped cell of varying length. The red circles, blue crosses and green asterisks ($3\text {d}$ only) correspond to computed effective diffusivities obtained by solving (8) on domains described in section A.1. The curves are least squares cubic polynomial fits using the average of the estimated diffusivities (Color figure online)

**Fig. 10:**
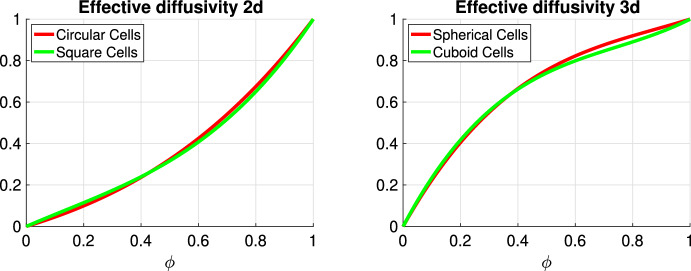
Comparison of the polynomials describing the effective diffusivity for the microstructures considered in sections 6.2 and A.1

**Fig. 11:**
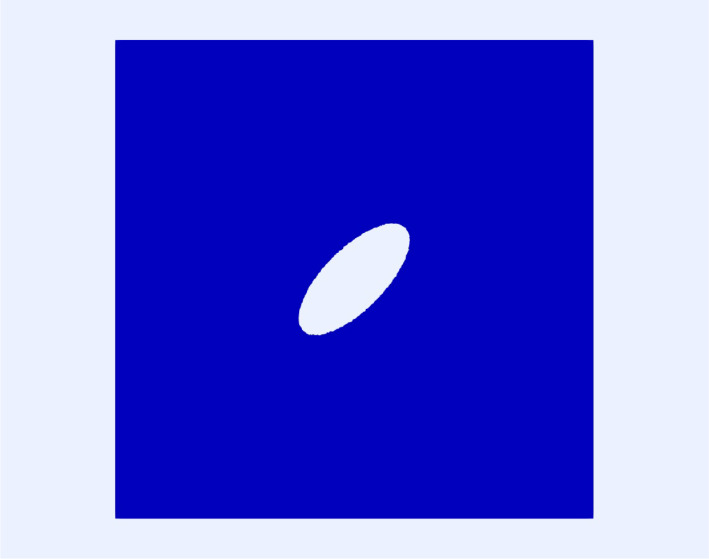
An example of a microscopic geometry that leads to a full diffusion tensor

**Fig. 12:**
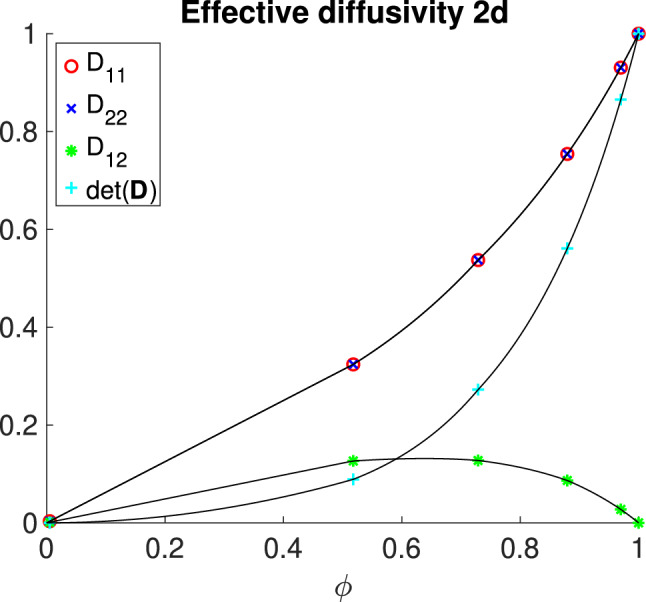
Fitted effective diffusivity in $2\textrm{d}$ for the example in section 7.1. The red circles, blue crosses and green asterisks correspond to computed effective diffusivities obtained by solving (8) on domains described in section 7.1. The curves for the diffusivities are interpolated values (Log-Euclidean for $\phi >0.518$, linear for $\phi <0.518$). The determinant is computed directly using the interpolated diffusivity values (Color figure online)

## Data Availability

Data sharing is not applicable to this article as no new data were created or analysed in this study.

## Appendix A. Additional computations of effective diffusion tensors

We report on computations of the effective diffusion tensor considering different microstructures.

### A.1. Alternative cell shape

In order to consider different cell shapes and allow smaller volume fractions of ECM to be modelled without issues arising due to meshing complex geometries we consider the following geometric setup for the microstructure. The domain corresponds to a region of the form $[-2,2]^d\setminus [-Y,Y]^d$ where $d=2,3$ and $Y=0.35n, n=1,2,3,4,5$, i.e., the domain is a hypercube of side length 4 with a single hypercube of side length 0.7*n* removed (i.e., the cell is a hypercube) both centred at the origin. The corresponding volume fractions of ECM are 0.97, 0.88, 0.72, 0.51 and 0.23 in $2\textrm{d}$ and 0.99, 0.96, 0.86, 0.66 and 0.33 in $3\textrm{d}$ for $n=1,2,3,4,5$ and 6 respectively. We approximate the solution to (8) using a finite element method implemented in the FEniCS software (Logg et al. 2012). We take $D_s(0)=0$ as a further fitting point as we assume the soluble MMPs do not diffuse through the cancer cells. Fitting a cubic polynomial in the $2\textrm{d}$ and $3\textrm{d}$ case (to the average of the computed diffusivities, noting that the off-diagonal components are zero) yields the following expressions

36 $$\begin{aligned} {D_s(\phi )}=\big (0.67\phi ^3-0.27\phi ^2+0.60\phi \big ){D_s(1)}, \end{aligned}$$

in $$2\textrm{d}$$ and

37 $$\begin{aligned} {D_s(\phi )}=\big (1.32\phi ^3-2.94\phi ^2+2.62\phi \big ){D_s(1)}, \end{aligned}$$

in $3\textrm{d}$. Figure 9 shows plots of the effective diffusivity as the volume fraction of ECM changes.

We note that in the relevant range of volume fractions, $\phi \in [0,1]$, the fitted polynomial for the effective diffusivity is very close to the previously obtained polynomials, see section 6.2, as shown in Figure 10.

### Microstructure that results in a full effective diffusion tensor

In this section we consider a microstructure that results in a full effective diffusion tensor, specifically the domains we use correspond to squares of the form $[0,4]^2$ with $n^2$, for $n=1,2,3,4$, ellipse(s) of major and minor axes 0.6 and 0.25 removed. In order to obtain a full effective diffusion tensor the orientation of the ellipses is such that the major axis of each ellipse is parallel to the line $y=x$. The centres of the ellipses are taken to form a square lattice, with the resulting geometry for a single ellipse as shown in Figure 11. The corresponding volume fractions of ECM are 0.970, 0.880, 0.729 and 0.518 for $n=1,2,3$ and 4 respectively.

For the choice of microscopic geometry considered here we note that, the effective diffusion tensor is not expected to be diagonal. This is reflected in the numerical results, for which an unstructured triangulation is used, in that the off diagonal components of the computed diffusion tensors for all geometries are of the same order of magnitude as the diagonal components. The diagonal components of the computed diffusion tensors on each specific geometry exhibit only small differences ($<1\%$). To obtain the effective diffusion tensor as a function of $\phi$ we note that a constraint we must enforce is that the diffusion tensor remains positive definite for all $\phi$ strictly positive. In higher spatial dimensions naive polynomial fits of the individual coefficients or polynomial interpolation do not guarantee this. We instead follow (Arsigny et al. 2006) and use Log-Euclidean interpolation to interpolate the tensor and also take ${\varvec{D}_s}_{i,j}(0)=0$, $i,j=1,2$ as a further fitting point since there is no diffusion of MMPs within the cells. As $\varvec{D}_s(0)$ is not strictly positive definite we use linear interpolation in the interval with $\phi =0$ as the left hand end point. For completeness we state the Log-Euclidean interpolation formula below and refer to Arsigny et al. (2006) for theoretical considerations. Given symmetric positive definite diffusion tensors $\varvec{D}(\phi _1)$ and $\varvec{D}(\phi _2)$ assuming without loss of generality that $0<\phi _1<\phi _2\le 1$ we set

$$ \varvec{L}_1=\operatorname {logm}(\varvec{D}(\phi _1))\quad \text {and}\quad \varvec{L}_2=\operatorname {logm}(\varvec{D}(\phi _2)) $$

and for $\phi \in [\phi _1,\phi _2]$ we set

$$ \varvec{L}(\phi )=\operatorname {expm}\left( \frac{(\phi _2-\phi )}{(\phi _2-\phi _1)}\varvec{L}_1+\frac{(\phi -\phi _1)}{(\phi _2-\phi _1)}\varvec{L}_2\right) , $$

where $\operatorname {logm}$ and $\operatorname {expm}$ denote the matrix logarithm and matrix exponential respectively. Figure 12 shows plots of the effective diffusivity as the volume fraction of ECM changes together with the interpolated values (Log-Euclidean for $\phi >0.518$ and linear for $\phi <0.518$).

We note that the diagonal components of the effective diffusivity appear to be a convex functions of the volume fraction whilst the off diagonal components appear to be concave (in 2d).
